# Isolation and Characterization of Equine Uterine Extracellular Vesicles: A Comparative Methodological Study

**DOI:** 10.3390/ijms22020979

**Published:** 2021-01-19

**Authors:** Carmen Almiñana, Alba Rudolf Vegas, Muhittin Tekin, Mubbashar Hassan, Rustem Uzbekov, Thomas Fröhlich, Heinrich Bollwein, Stefan Bauersachs

**Affiliations:** 1 Functional Genomics Group, Institute of Veterinary Anatomy, Vetsuisse Faculty Zurich, University of Zurich, 8315 Lindau, Switzerland; alba.rudolfvegas@uzh.ch (A.R.V.); stefan.bauersachs@uzh.ch (S.B.); 2 UMR85 PRC, INRAE, CNRS 7247, Université de Tours, IFCE, 37380 Nouzilly, France; 3 Clinic of Reproductive Medicine, Department for Farm Animals, Vetsuisse-Faculty, University of Zurich, 8057 Zurich, Switzerland; Muhittin.Tekin@uzh.ch (M.T.); mubbashar.hassan@uvas.edu.pk (M.H.); hbollwein@vetclinics.uzh.ch (H.B.); 4 Laboratoire Biologie Cellulaire et Microscopie Electronique, Faculté de Médecine, Université de Tours, 37032 Tours, France; rustem.uzbekov@univ-tours.fr; 5 Faculty of Bioengineering and Bioinformatics, Moscow State University, 119992 Moscow, Russia; 6 Gene Center, Laboratory for Functional Genome Analysis, LMU Munich, 81377 Munich, Germany; frohlich@genzentrum.lmu.de

**Keywords:** extracellular vesicles, exosomes, uterus, mare, *Equus caballus*, EVs protein cargo, mass spectrometry, uterine fluid, uterine lavage

## Abstract

Extracellular vesicles (EVs) have been identified in the uterine fluid in different species and have been pointed as key players in the embryo-maternal dialogue, maternal recognition of pregnancy and establishment of pregnancy. However, little is known about the uterine EVs in the mare. Therefore, the present study aimed at characterizing EVs from uterine lavage of cyclic mares by comparing five EVs isolation methods and the combination of them: (1) ultracentrifugation (UC); (2) concentration of lavage volume by Centricon ultrafiltration (CE); (3) the use of CE with different washing steps (phosphate-buffered saline with or without trehalose); (4) size-exclusion chromatography with iZON-qEV columns, and (5) a combination of the methods with best results based on EVs yield, purity, and protein cargo profiles. Transmission electron microscopy and Western blotting confirmed the isolation of EVs by all methods but with quantitative and qualitative differences. Mass spectrometry provided differences in protein profiles between methods, number of identified proteins, and protein classes. Our results indicate that the combination of CE/trehalose/iZON/UC is an optimal method to isolate equine uterine EVs with good yield and purity that can be applied in future studies to determine the role of equine uterine EVs in embryo-maternal interactions.

## 1. Introduction

Extracellular vesicles (EVs), including exosomes and microvesicles (MVs), are small cell-derived membrane vesicles that can be detected in all biological fluids as a result of membrane shedding by any cell type in the organism [1,2,3,4]. Exosomes (30–150 nm) have an endocytotic origin, released upon fusion of a multi-vesicular body with the cell membrane, while microvesicles (100–1000 nm) bud directly from the cell membrane [5]. Among their interesting features, EVs are carriers of a wide range of biologically active molecules (RNAs, proteins, lipids, metabolites, genomic DNA), which can traffic to local or distant target cells, where they can execute defined biological functions by transferring this cargo cell-to-cell [6]. Therefore, the role of these EVs as mediators of cell-to-cell communication is well recognized to date [7,8]. Besides, novel biological functions of EVs released under different physiological or pathological conditions are continuously being described, highlighting their importance in different fields and pointing them as unique diagnostic tools with a significant therapeutic potential as well [9]. 

Given that gamete/embryo-maternal communication has been defined as a determinant of reproductive success in many species, including human [10], studying the role of EVs present in reproductive biological fluids (follicular, oviductal, uterine fluids) has become a hot topic in the last few years [11,12,13,14,15,16,17,18]. Extracellular vesicles identified in the oviduct and in the uterus of different species have emerged as key mediators of the early reproductive events contributing to successful pregnancy [19,20,21] and as potential biomarkers of reproductive health and disease [22]. 

In the mare, little is known about the uterine EVs and their contribution to embryo-maternal interactions leading to successful pregnancy. Due to the very unique features of reproduction in equids, current results from other mammalian species such as mouse, sheep, bovine, pig or human [14,23,24,25], cannot be simply applied to horses. Studies in the mare have focused on EVs from follicular fluid and their miRNAs and protein cargo, as a possible new form of cell communication within the ovarian follicle [26]. Furthermore, circulating EVs in the serum of pregnant and cyclic mares on days 12, 14, 16, and 18 post-ovulation have been identified, and differential exosomal miRNA cargo has been found among pregnant and non-pregnant animals [27]. The lack of uterine EVs studies in the mare compared to other species might be related to: (i) the considerable volume of uterine lavage as starting material; (ii) the challenge of concentrating this volume without substantial loss of EVs and; (iii) the lack of a protocol assuring the isolation of equine uterine EVs with considerable purity that allows subsequent analysis of the diverse EVs molecular cargo and the functional impact on the embryo or embryo-maternal interactions. 

Therefore, in this study we aimed at establishing a protocol to isolate equine uterine EVs from uterine lavages by comparing five different isolation methods and considering the influence of the individual variability of the mares. Given that the isolation protocol has profound effects on the omics results applied post-isolation (e.g., protein or RNA profiles of EVs) [28,29], we aimed also at analyzing the protein composition of EVs obtained by different methods by mass spectrometry. Furthermore, a comparative analysis of uterine EVs protein cargo among species based on available data was performed in attempt to discern between species or methods differences. The methodological study presented here will show the variability among isolation methods and will provide the first characterization of equine uterine EVs, which is an important step towards the study of the role of EVs in embryo-maternal communication in utero in the mare.

## 2. Results

### 2.1. Isolation and Characterization of Equine Uterine EVs by Different Methods

#### 2.1.1. TEM Observations and EVs Size Distribution

**Figure 1:**
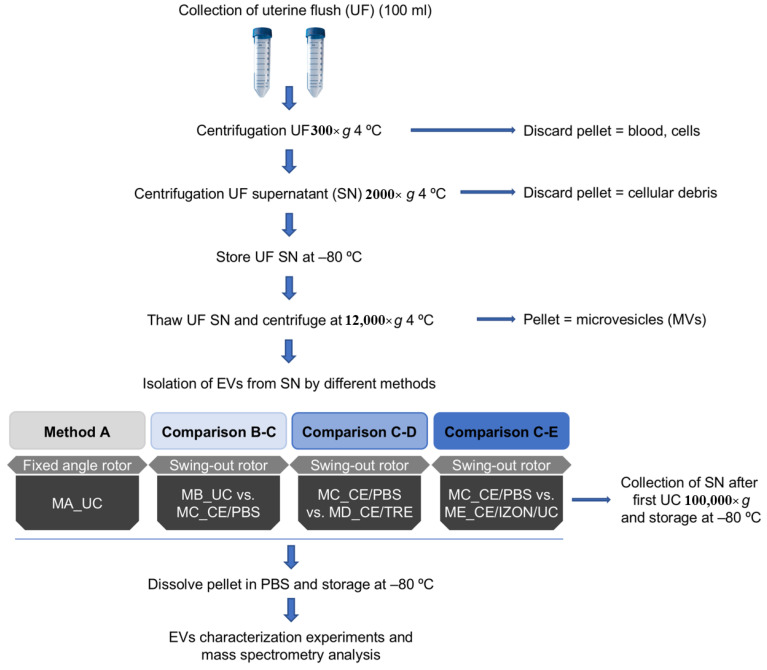
Schematic representation of the different protocols tested for extracellular vesicles (EVs) isolation from equine uterine fluid. Method A: By ultracentrifugation (UC) 100,000× *g* (2×) 90 min using fixed angle Beckman rotor (MLA-55) and Beckman bottles (No. 355603) (MA_UC). Method B: UC with swing-out rotor MLS-50 (MB_UC). Method C: ultrafiltration by using Centricon filters (CE) followed by UC with swing-out rotor (MC_CE/PBS). Method D: ultrafiltration using CE with a washing step with PBS/25 mM trehalose (TRE) followed by UC (MD_CE/TRE). Method E: Combination of CE, followed by size exclusion chromatography (Izon qEV columns) and the collection of fractions enriched in EVs (7,8,9) and concentration of EVs by UC (ME_CE/IZON/UC).

**Figure 2:**
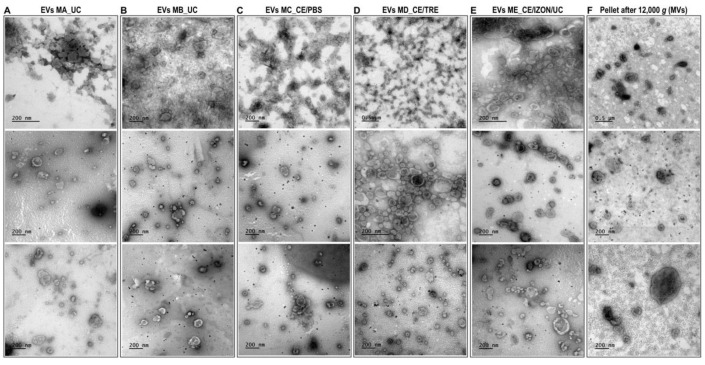
Representative images of extracellular vesicles (EVs) and microvesicles (MVs) isolated by different methods obtained by transmission electron microscopy observations. (**A**) EVs obtained by Method A, ultracentrifugation (UC) 100,000× *g* (2×) 90 min using fixed angle Beckman rotor (MLA-55) and Beckman bottles (No. 355603); (**B**) EVs obtained by Method B, UC with swing-out rotor MLS-50; (**C**) EVs obtained by Method C, ultrafiltration using Centricon filters (CE) followed by a washing step with PBS and then UC; (**D**) EVs obtained by Method D, similar to C but washing step with PBS/25 mM trehalose; (**E**) EVs obtained by Method E, a combination of CE, followed by size exclusion chromatography (Izon qEV columns) and concentration of EVs fractions by UC; and (**F**) Microvesicles obtained from the pellet after centrifugation at 12,000× *g* for 30 min.

**Figure 3:**
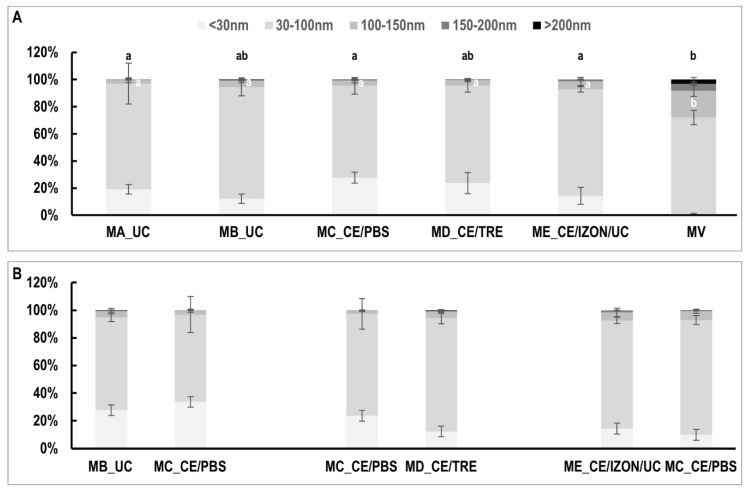
Comparison of extracellular vesicles (EVs) size distribution among isolation methods. (**A**) EVs size distribution among all different EVs isolation methods examined. (**B**) Two-by-two comparisons of EVs size distribution of methods MB with MC, MC with MD, and MC with ME, using uterine lavage samples from the same mares for each comparison. MA_UC: EVs obtained by Method A, ultracentrifugation (UC) 100,000× *g* (2×) 90 min using fixed angle rotor and Beckman bottles; MB_UC: UC with swing-out rotor and Beckman tubes; MC_CE/PBS: ultrafiltration by using Centricon filters followed by a washing step with PBS; MD_CE/TRE: similar to C but washing step with PBS/25 mM trehalose; ME_CE/IZON/UC: EVs obtained by the combination of CE, followed by SEC, Izon qEV columns and concentration of EVs fractions by UC; MVs: microvesicles obtained after centrifugation at 12,000× *g* for 30 min. Data are represented as mean with the error bars, indicating the standard error of the mean (SEM). Different letters indicate significant differences among EVs isolation methods within a specific EVs size range (100–150 and >200) (a,b: *p* < 0.05).

A total of 12 EVs sample preparations were used for TEM observations to evaluate the presence of EVs obtained by five different methods (MA-ME, see Figure 1) (three replicates/isolation methods). Moreover, pellets obtained after centrifugation at 12,000× *g* containing microvesicles (MVs) were examined (three replicates) by TEM. All different EVs isolation protocols permitted the isolation of EVs as observed by TEM (Figure 2), although quantitative and qualitative differences were observed among methods in terms of preservation of EVs morphology, EVs aggregates, impurities in the sample (Figure 2), or differences in size distribution (Figure 3). 

In all EVs preparations analyzed, TEM observations showed a population of small EVs (30–100 nm) resembling exosomes and a population of large EVs (>100 nm) resembling MVs (Figure 2). According to the literature, MVs range from >100 up to ∼1000 nm [30]. In the pellets obtained after centrifugation of the uterine flush at 12,000× *g* (Figure 2F), a bigger proportion of MVs was found compared to EVs samples (Figure 2F). Histograms of Figure 3A show the distribution of EVs obtained from the different methods compared two-by-two (MB_UC vs. MC_CE/PBS; MC_CE/PBS vs. MD_CE/TRE; MC_CE/PBS vs. ME_CE/Izon/UC) (Figure 3A), with no significant differences between methods. When the EVs size distribution was analyzed among all the EVs preparations obtained from different EVs methods including MVs preparations (Figure 3B), the percentage of vesicles with 100–150 nm size range was significantly higher in MVs preparations (19.43 ± 1.6%) than in all EVs samples no matter the method used (2.7–5.8% range among all methods). Similarly, the percentage of vesicles with >200 nm size range was significantly higher in MVs preparations (3.28 ± 1.6%) than in EVs samples obtained by methods MA_UC, MC_CE/PBS and ME/CE/Izon/UC (0 ± 0%; 0.13 ± 0.08% and 0 ± 0%, respectively).

Regarding the qualitative differences, the use of CE filter with trehalose (MD_CE/TRE) seems to preserve more the morphology of EVs as observed by TEM (Figure 2D). Furthermore, the average percentage of EVs with range 30–100 nm (exosomes) was higher in MD_CE/TRE (82.2 ± 4.1%, *n* = 3) compared to MC_CE/PBS (73.8 ± 11%, *n* = 3) and a lower percentage of very small EVs < 30 nm was found in MD_CE/TRE (12.2 ± 3.3%, *n* = 3) compared to MC_CE/PBS (23.6 ± 10.5%, *n* = 3) (Figure 3A), although these differences were not statistically significant (*p* value for EVs 30–100 nm: 0.557; *p* value for EVs < 30 nm 0.462). On the other hand, the use of method ME_CE/IZON/UC seemed to provide samples with less impurities and EVs aggregates (Figure 2E) and without vesicles > 200 nm compared to MC_CE/PBS (Figure 3A).

#### 2.1.2. Protein Profile of EVs by SDS-PAGE and Immunoblotting for Known Exosomal Markers

First, the protein profiles of EVs (after 100,000× *g*) and MVs (after 12,000× *g*) from the same samples for three different biological preparations (UF from three mares) were examined by SDS-PAGE (Figure S1). Although the same amount of protein based on BCA measurements of EVs and MVs samples was used for the three preparations, individual variations mainly in replicate 3 compared to 1 and 2 were observed (Figure S1).

**Figure 4:**
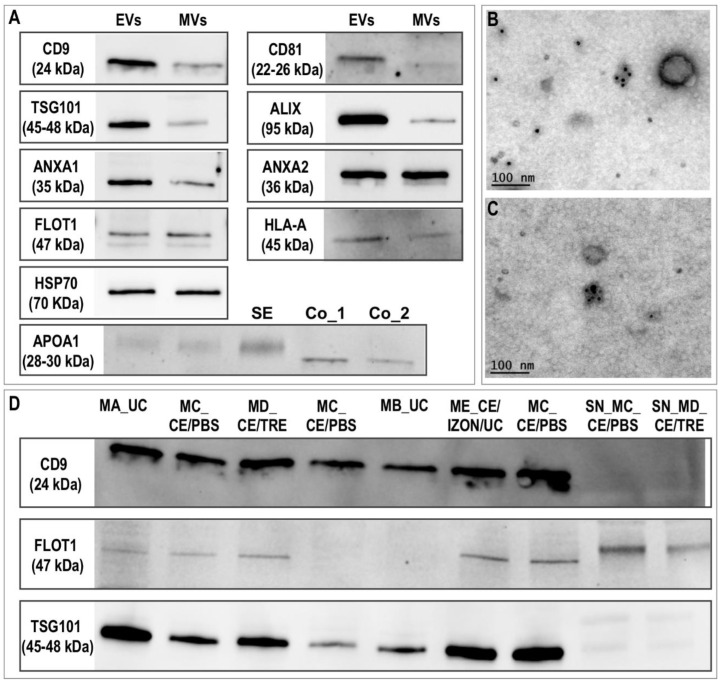
Characterization of equine uterine extracellular vesicles (EVs) protein profile. (**A**) Western blotting characterization of EVs and microvesicles (MVs; pellet after centrifugation of uterine lavage at 12,000× *g*) for known exosomal protein markers and apolipoprotein A1 (SE: Serum from mares; Co_1 and Co_2: Human apolipoprotein protein control 0.5 and 0.2 µg). (**B**,**C**) Representative images of electron microphotographs of CD9 immunogold labelling of EVs. (**D**) Western blotting comparison of EVs obtained by different methods for three known exosomal markers (CD9, FLOT1, and TSG101). MA_UC: EVs obtained by ultracentrifugation (UC) 100,000× *g* (2×) 90 min using fixed angle rotor and Beckman bottles; MB_UC: UC with swing-out rotor and Beckman tubes; MC_CE/PBS: ultrafiltration by using Centricon filters followed by a washing step with PBS; MD_CE/TRE: similar to before but washing step with PBS/25 mM trehalose; ME_CE/IZON/UC: obtained by the combination of CE, followed by SEC, Izon qEV columns and the collection of fractions enriched in EVs (7,8,9) and concentration of EVs fractions by UC; SN_MC_CE_PBS and SN_MD_CE_TRE: supernatant obtained after first UC from MC_CE/PBS and MD_CE/TRE.

Immunoblotting results showed that EVs were positive for known exosomal markers (tetraspanins: CD9, CD81; and also, TSG101, ALIX (PDCD6IP), flotillin-1 (FLOT1), ANXA1, ANXA2, HSP70 (HSPA1A/HSPA1B), HLA-A) (Figure 4A). For almost all the markers tested, much stronger bands were found in EVs preparations compared to MVs, except for HSP70 and ANXA2, where similar band intensity was found. These results are in line with TEM observations, since vesicles resembling exosomes (30–100 nm) were also found in MVs preparations, although in lower concentrations. Furthermore, immunogold labelling in TEM for the tetraspanin CD9, revealed that vesicles in EVs preparations were positive for CD9 (Figure 4B,C). 

Additionally, as a negative marker, apolipoprotein A1 (APOA1) was examined in preparations of EVs and MVs, as well as in serum from mares and in a positive control (apolipropotein A1, Antibodies-online GmbH, Germany, ABIN934456 Human APOA1). Very faint bands were observed in EVs and MVs for APOA1 compared to equine serum or controls (Figure 4A).

To examine differences among EVs isolation methods, three exosomal markers (CD9, TSG101 and FLOT1) were tested in EVs obtained by all the different methods by immunoblotting. Figure 4D shows that all methods derived EVs that were positive for CD9, TSG101 and FLOT1 with slight differences among the methods, which could also be due to the use of different biological samples. The supernatant from two different methods was also examined in these immunoblottings for the same exosomal markers, showing that these proteins were present in the EVs and not in the supernatant (SN) or in a very small amount (Figure 4D).

### 2.2. Protein and RNA Cargo Profiles of EVs Obtained by Different Isolation Methods

#### 2.2.1. Differential Protein Concentration among Isolation Methods

**Figure 5:**
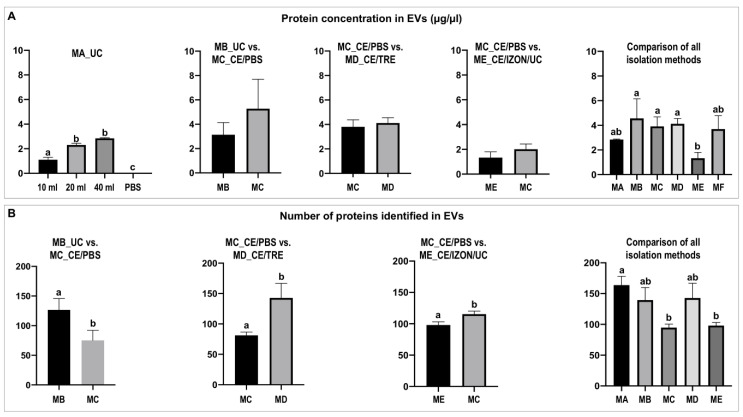
Protein concentration and number of proteins identified in equine uterine extracellular vesicles (EVs). (**A**) Comparison of protein concentration (µg/µL) of EVs isolated by different methods. (**B**) Comparison of the number of proteins identified in EVs by mass spectrometry among methods. Different letters indicate significant differences (a,b: *p* < 0.05). EVs isolation methods: MA_UC: EVs obtained by ultracentrifugation (UC) using Beckman fixed angle rotor from 10, 20, and 40 mL of uterine flush, and from 10 mL PBS as a control; MB_UC: EVs obtained by Beckman swing-out rotor; MC_CE/PBS: EVs obtained by ultrafiltration using Centricon filters (CE), wash step with PBS and followed by UC swing-out rotor; MD_CE/TRE: similar to before but wash step with PBS/25 mM trehalose; ME_CE/IZON/UC: EVs obtained by combination of CE, followed by SEC, Izon qEV column and collection of fractions enriched in EVs (7,8,9) and UC swing-out rotor; and MF (MF_CE/TRE_IZON/UC): CE with PBS/25 mM trehalose wash step, Izon qEV columns and UC.

First, a proxy quantification of EVs was performed based on protein concentration as advised by the MISEV 2018 guidelines [31]. As stated above, method 1 was used to this end, by examining if EVs protein concentration increased as the uterine lavage sample volume increased. Method MA_UC in Figure 5A shows that the EVs protein concentration increased with sample volume, with significant differences between 10 and 20/40 mL (*p* < 0.05) and a tendency between 20 and 40 mL (*p* = 0.06). As expected, a protein concentration close to 0 was obtained from the PBS control (10 mL).

Furthermore, protein concentration was compared two-by-two for MC_CE/PBS vs. MB_UC, MD_CE/TRE vs. MC_CE/PBS, and ME_CE/Izon/UC vs. MC_CE/PBS, with no statistically significant differences found for each comparison (Figure 5A). However, it is to be noticed that for the comparison of methods E and C (ME_CE/IZON/UC vs. MC_CE/PBS) the obtained EVs samples showed lower protein concentrations than the other methods/comparisons (Figure 5A).

Consequently, a sixth method (MF) based on the obtained results for the different methods was used and combined CE/TRE, Izon-qEV, and UC. Figure 5A shows the comparison of protein concentration among all EVs isolation methods tested (MA-MF). Statistically significant differences were found in terms of protein concentration (Figure 5A) between ME_CE/IZON/UC (E) vs. MB_UC (B) (*p* = 0.0241); ME_CE/IZON/UC (E) vs. MC_CE/PBS (C) (*p* = 0.0165), and between ME_CE/IZON/UC (E) vs. MD_CE/TRE (D) (*p* = 0.0073). Additionally, differences in protein concentration were found also between ME_CE/IZON/UC (E) and MF_CE/TRE/IZON/UC (F) (*p* = 0.0597), although not statistically significant (Figure 5A).

#### 2.2.2. Differential Protein Cargo among Isolation Methods

A total of 1143 proteins contained in EVs samples were identified and quantified by mass spectrometry (Supplementary Tables S1 and S2). Mass spectrometry results indicated differences among EVs samples obtained by different isolation methods. First, differences were observed in the number of identified proteins among the different methods used (Figure 5B). For each pairwise comparison as well as when EVs from all methods were compared, statistically significant differences were observed among methods. These results revealed that the use of ultrafiltration (CE) or CE in combination with SEC Izon columns decreased the number of proteins identified but when CE was performed with PBS/TRE the number of proteins increased to similar values as the methods without ultrafiltration such as MA_UC or MB_UC (Figure 5B). The number of identified proteins for each method and the variability among replicates is shown in Supplementary Table S3.

**Figure 6:**
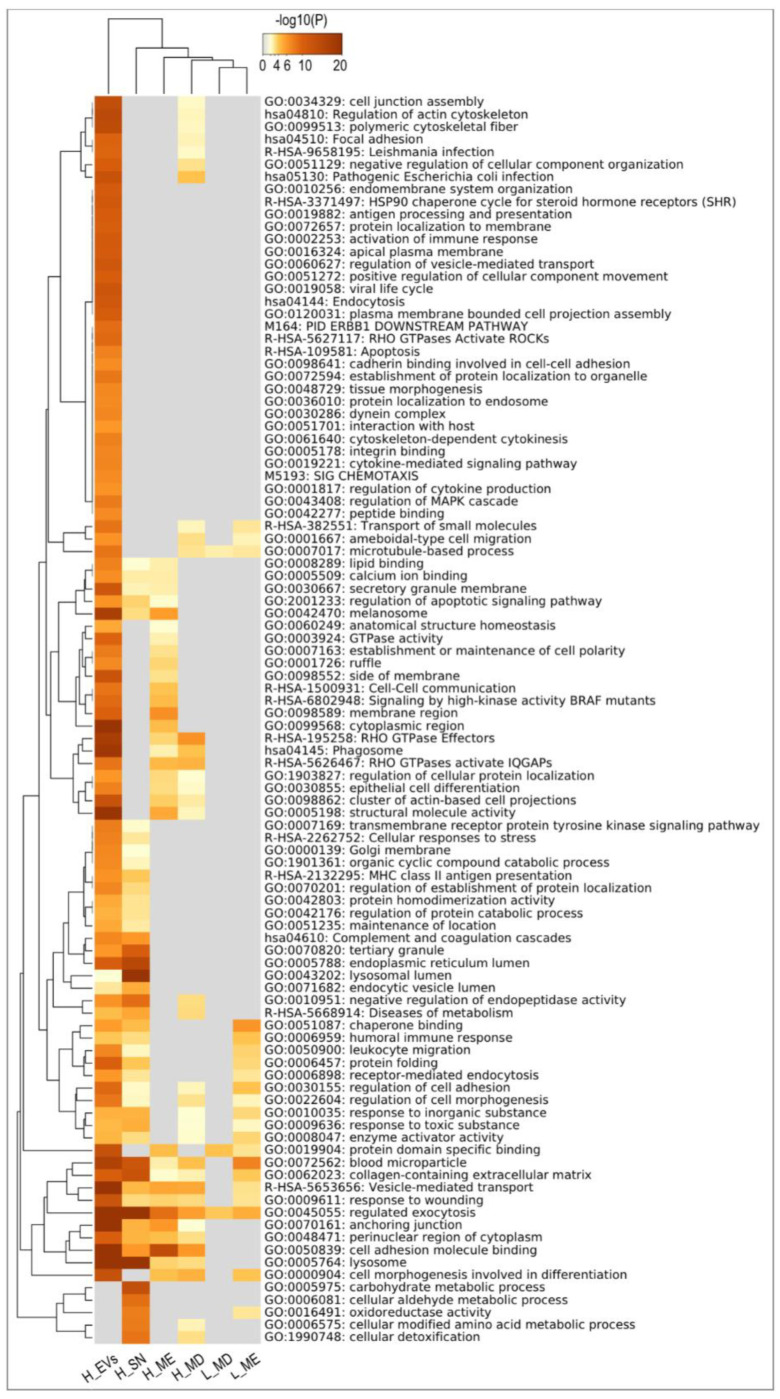
Functional enrichment analysis for proteins identified by mass spectrometry using the Metascape tool. Heatmap visualization of selected enriched terms across protein lists categorized as follows: (H_EVs) proteins highly enriched in EVs obtained by all methods; (H_MD) proteins highly abundant in MD_CE/TRE; (H_ME) proteins highly abundant in ME_CE/IZON/UC; (L_MD) proteins low abundant in MD_CE/TRE; (L_ME) proteins low abundant in ME_CE/IZON/UC; and (H_SN) proteins highly enriched in supernatant (SN). Bar graph of enriched terms across differentially abundant proteins colored by *p*-values representing enriched clusters up to a score of 2.

Additionally, all the identified proteins by mass spectrometry were grouped based on high or low abundancy in EVs in comparison to supernatant (SN, free proteins) as follows (for definition of these protein lists, see Methods 4.4.4): proteins higher in EVs compared to supernatant (H_EVs; 606) and proteins higher in supernatant (H_SN; 155) (Supplementary Table S4). Furthermore, lists were generated for proteins higher in MD_CE/TRE vs. MC_CE/PBS (H_MD; 36), proteins higher in ME_CE/IZON/UC vs. MC_CE/PBS (H_ME; 45), proteins lower in MD_CE/TRE (L_MD;10), and proteins lower in ME_CE/IZON/UC (L_ME; 17) (Supplementary Table S5). These six sets of proteins (H_EVs; H_SN; H_MD; L_MD; H_ME; L_ME) were used for functional enrichment analysis with the Metascape tool (Supplementary Tables S6–S8). In Figure 6, a Metascape heatmap plot represents the top clusters of enriched functional terms for the six different sets of proteins (100 functional annotation clusters). In the heatmap, each row represents one enriched cluster, the color scale represents statistical significance, with gray color indicating lack of significance. Figure 6 shows functional categories in common to H_EVs and H_SN or exclusively in H_EVs (24 clusters, e.g., GO:0060627 regulation of vesicle-mediated transport; GO:0002253 activation of immune response; ko04144: Endocytosis; GO:0034329: cell-cell junction assembly; R-HAS-109581: Apoptosis; all categories and log *p*-values in Supplementary Table S8).

**Figure 7:**
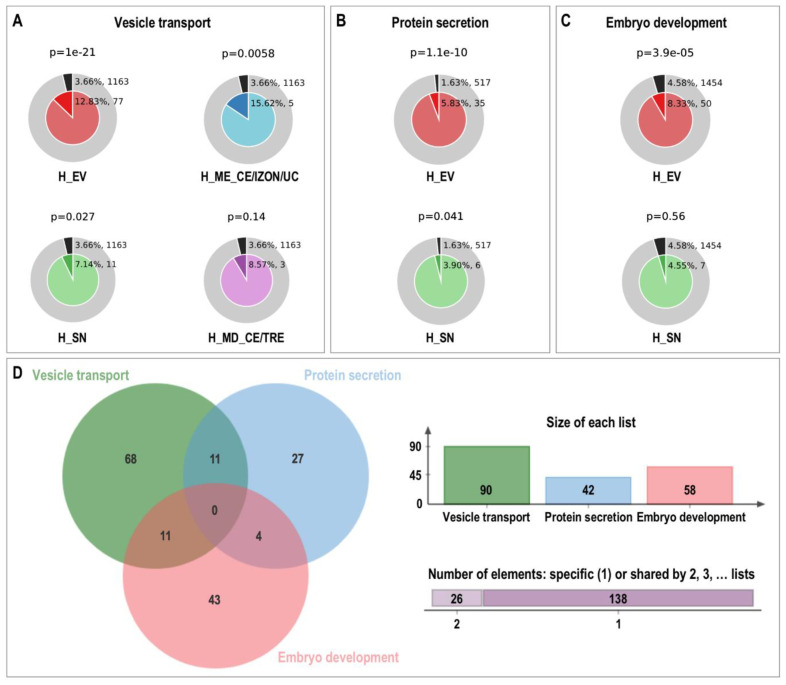
Functional membership analysis for proteins identified by Mass Spectrometry for specific terms using Metascape tool. Enrichment of proteins matching membership terms: (**A**) “vesicle transport”, (**B**) “protein secretion” and (**C**) “embryo development”. The outer pie shows the number and the percentage of proteins in the background that are associated with the membership (in black); the inner pies shows the number and the percentage of proteins in the individual input gene list that are associated with the membership. The *p*-value on the top of the pie charts indicates whether the membership is statistically significantly enriched in the list. H_EV: proteins highly enriched in EVs obtained by all methods; H_SN: proteins highly enriched in supernatant (SN); H_MD_CE/TRE proteins highly abundant in MD_CE/TRE; H_ME_CE/IZON/UC: proteins highly abundant in ME_CE/IZON/UC. (**D**) The Venn diagram illustrates the total number of proteins found for each membership term using the Metascape tool and the number of protein common or exclusive for each term.

Furthermore, using Metascape Membership tool, enrichment of genes in terms matching keywords such as, “vesicle transport”, “protein secretion” and “embryo development” were searched. Figure 7 shows the results of the membership analysis for these three keywords for the proteins grouped in H_EVs, H_SN, H_MD_CE/TRE and H_ME_CE/IZON/UC. The assigned proteins and the specific functional terms can be found in Supplementary Table S9. The outer ring of each pie (grey) shows the number and the percentage of genes in the background that are associated with the membership term(s) (in black). While the inner of each pie shows the number and the percentage of genes in the individual input gene list that are associated with the membership term. The *p*-value at the top of each pie indicates whether the membership term is statistically significantly enriched in the list. Membership term analysis for “vesicle transport” showed that a good number of the proteins identified in H_EVs (12.83%, 77 proteins) were related to corresponding GO terms, with a higher number of proteins than expected by chance also for H_ME_CE/IZON/UC (15.62%, five proteins) and H_MD_CE/TRE (8.57%, three proteins). Regarding the membership term “protein secretion”, 5.83% (35) of the proteins in H_EVs were related to this term, while 3.90% (6) for H_SN. For the “embryo development” term, 8.33% (50) of proteins in H_EVs and only 4.55% (7) in H_SN were found. In Figure 7D, the Venn diagram illustrates the total number of proteins found for each keyword using the Metascape tool and the number of proteins that are common or exclusive for each term (“vesicle transport”, “protein secretion”, and “embryo development” Supplementary Table S9). Metascape Enrichment Membership analysis showed functional terms for 58 identified proteins in EVs related to in utero embryonic development (GO:0001701) and embryo development (GO:0009790), as for example, FN1, FURIN, LAMB2, MTHFD1 and MYH9 (Supplementary Table S9).

To provide an overview of the proteins identified in EVs among methods, we focus particularly on the proteins identified in MD (CE with PBS/trehalose) and ME (Izon SEC) methods with differences compared to MC. Supplementary Figure S2 and Supplementary Table S10 illustrate these selected proteins and their associated intensity values that showed differences in EVs protein abundance among isolation methods. In addition, intensity values for the supernatant samples after first UC (SN, methods MC and MD) are shown to see if these proteins are also found in the SN and with which intensity. For example, valosin containing protein (VCP), furin (FURIN), methylenetetrahydrofolate dehydrogenase (MTHFD1) and mucin-4 (MUC4) were only identified in EVs by all methods but not in SN samples. By contrast, SPINK7 was almost exclusively present in SN samples. P19 lipocalin (P19) was present in both EVs isolated by all methods and SN. Regarding the variability among methods, calmodulin (CALM1), ATPase H^+^ transporting V1 (ATP6V1G1), laminin subunit beta 2 (LAMB2), semaphorin 4B (SEMA4B) and p21 (RAC1) activated kinase 2 (PAK2) were identified when MD_CE/TRE was used, while coactosin such as F-actin binding protein 1 (COTL1), tubulin beta class I (TUBB), chloride intracellular channel 4 (CLIC4), and envoplakin (EVPL) were identified when SEC iZON qEV (ME) was used. By contrast, stanniocalcin 1 (STC1) and alcohol dehydrogenase 5 (ADH5) were not identified when purification with SEC iZON qEV (ME) was used, but they were identified when other methods where used.

Additionally, in search of differences in protein patterns among methods, pairwise quantitative analyses point to several differential EVs proteins among isolation methods (*p*-value < 0.1) for MB vs. MC (seven proteins, e.g., VCP); MC vs. MD (16, e.g., PAK2, SEMA4B, and MMP26), and ME vs. MC (18, e.g., TUBB, FURIN, and MMP26). Mean-centered log2 intensity values of these proteins are illustrated in the heatmaps in Figure S3. These results indicate that the use of different EVs isolation methods leads to the identification of a different EVs protein cargo. The complete list of proteins detected for each pairwise comparison can be found in Supplementary Tables S11–S13.

#### 2.2.3. Differential EVs RNA Concentration Obtained by Different Isolation Methods

**Figure 8:**
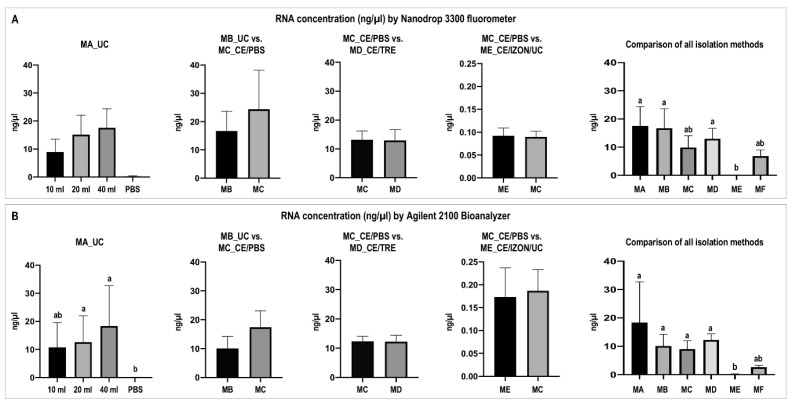
RNA concentration of equine uterine extracellular vesicles (EVs) isolated by different methods. (**A**) RNA concentration of EVs measured by Nanodrop 3300 fluorometer. (**B**) RNA concentration of EVs measured by Agilent 2100 Bioanalyzer. Different letters indicate significant differences among EVs isolation methods. (a,b: *p* < 0.05). Method MA_UC: EVs obtained by ultracentrifugation (UC) using fixed angle rotor starting from 10, 20, and 40 mL uterine lavage sample or using PBS as a control; MB_UC: EVs obtained by UC with swing-out rotor; MC_CE/PBS: ultrafiltration by using Centricon filters and subsequent UC with swing-out rotor; MD_CE/TRE: EVs obtained by ultrafiltration with washing step with PBS/25mM trehalose and subsequent UC with swing-out rotor; ME_CE/IZON/UC: EVs obtained by ultrafiltration followed by size exclusion chromatography (Izon qEV columns, collection of fractions 7,8,9 enriched in EVs and concentration of EVs fractions by UC swing-out rotor; MF_CE/TRE/IZON/UC: CE with PBS/25 mM trehalose wash step, Izon qEV columns and UC.

First, a proxy quantification of EVs was also performed based on RNA concentration and using EVs obtained by MA_UC. Method MA_UC graphs in Figure 8A,B shows that the EVs RNA concentration had a tendency to increase with the volume used for EVs isolation, measured by Nanodrop 3300 fluorometer and Bioanalyzer, respectively. As expected, RNA concentrations close to 0 were obtained from the PBS control (no presence of EVs). While no statistical differences were found in the two-by-two comparisons, it is to be noted that that in the comparison C–E (ME_CE/IZON/UC vs. MC_CE/PBS), much lower RNA concentrations were obtained from isolated EVs from both methods compared to B–C and C–D comparisons (Figure 8A,B), similarly to protein concentration results (Figure 5). However, when ultrafiltration (MC_CE/PBS) was compared to MB_UC in comparison B-C, no significant differences in RNA concentration were observed, as observed for the protein in the comparison MC vs. MB. When all methods were compared, MC_CE/PBS provided similar RNA concentration results compared to other methods except to ME_CE/IZON/UC, which was significantly different (*p* value = 0.0214 for Bioanalyzer) (Figure 8A,B). However, when CE/TRE was combined with iZON qEV in MF the RNA concentration showed similar values to other methods (Figure 8A,B), as well as the protein concentration results.

#### 2.2.4. RNase Protective Assay: Ascertaining that the Isolated RNA Is Only Confined within EVs

**Figure 9:**
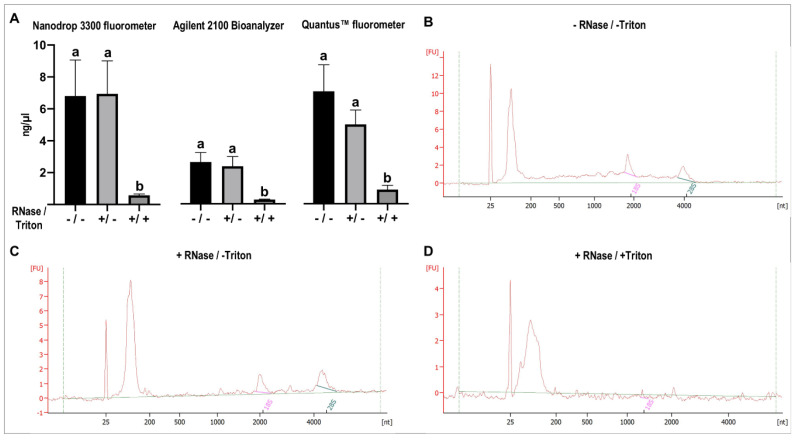
RNA quality and quantity of equine uterine extracellular vesicles (EVs) after RNase protective assay. EVs samples were treated with RNase (+/−), RNase and Triton X-100 (+/+) or not treated (−/−). (**A**) RNA was isolated and measured with Nanodrop 3300 fluorometer, Agilent 2100 Bioanalyzer, and Quantus™ fluorometer. Different letters indicate significant differences among RNA treatments (a,b: *p* < 0.05). Bioanalyzer profiles show RNA quality assessment of not treated EVs samples (**B**); EVs samples treated with RNase but not Triton X-100 (**C**); and EVs samples treated with RNase and Triton X-100 before RNA isolation and quantification (**D**).

In Figure 9, concentrations of RNA of EVs isolated with method MF_CE/TRE/IZON/UC subjected to different treatments (RNase A; RNase A combined with Triton X-100 and untreated samples) and measured by three different methods (Nanodrop 3300 fluorometer, Agilent 2100 Bioanalyzer, and Quantus™ fluorometer) are represented and show a slight variation in RNA concentration values among measurement methods. RNA quantification of the three treatments showed that no significant differences were found between untreated samples and samples treated with RNase (Figure 9A). By contrast, when Triton X-100 was used in combination with RNase, RNA in all samples was almost totally degraded, as shown in the Bioanalyzer profiles in Figure 9D. Bioanalyzer profiles of untreated samples and samples treated only with RNase, showed similar profiles with three RNA peaks representing small RNAs, and 18S and 28S ribosomal RNAs, the latter relatively low compared to the smaller RNA fragments (Figure 9A,B). These RNA profiles indicate that smaller RNAs are dominant in equine uterine EVs RNAs.

#### 2.2.5. Comparative Analysis of Uterine EVs Protein Cargo among Species

**Figure 10:**
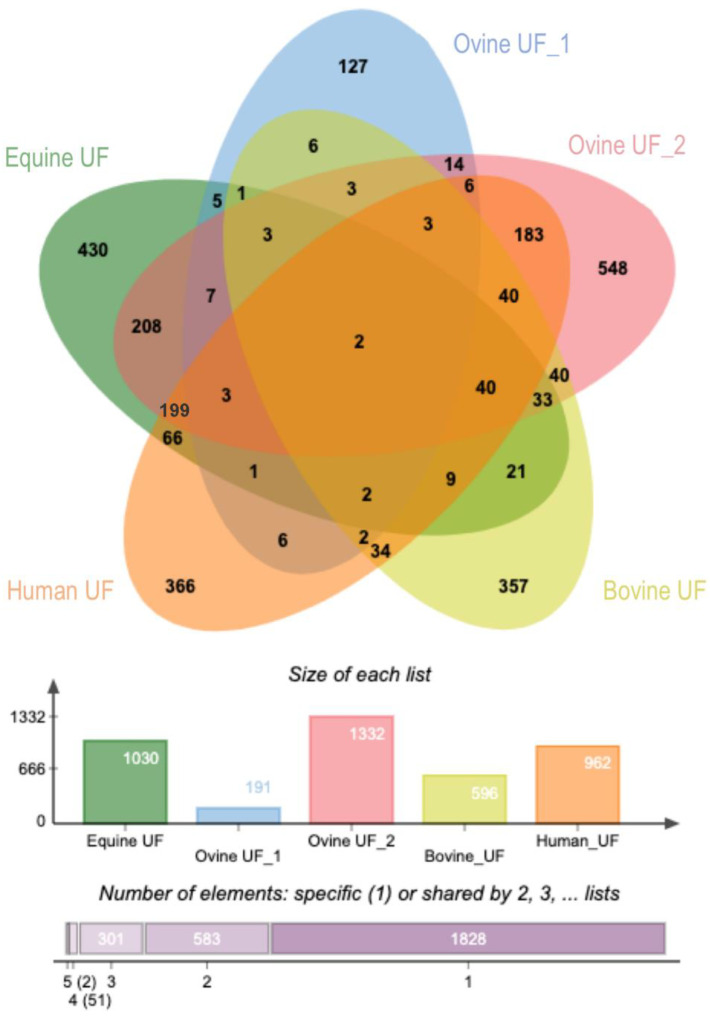
Comparative analysis of uterine EVs protein cargo among species. Venn diagram showing the overlap of proteins identified in uterine EVs from uterine flushings collected in different species and studies using different EVs isolation methods (number of total proteins).

Figure 10 shows the overlap of identified proteins (proteins with official gene symbol) among five studies including our study (equine, ovine, bovine, and human) [12,18,32,33]. The percentage of overlap among species and the EVs isolation method used in each study is shown in Table 1. It was observed that only two proteins were in common among the five studies in four different species (ATP1A1; YWHAZ). Moreover, this comparison showed big differences between studies in the same species performed under similar conditions by the same authors (Ovine UF_1 vs. Ovine UF_2) (3% overlap) but different isolating methods and thus, highlighting the importance of the method and its effect on the identified proteins. The highest similarities were found between equine uterine EVs (present study) and ovine uterine EVs (Uterine_UF_2; from O´Neil et al., 2020) (48%) and between the latter and human uterine EVs (45%). By contrast, the lowest similarities were found between Ovine UF_1 study and the rest of the studies (2–21%).

## 3. Discussion

In the present study, equine uterine EVs derived from uterine lavages from cyclic mares were isolated by using different EVs isolation methods. Although with all methods tested EVs were obtained, we found qualitative and quantitative differences in EVs preparations. Our results highlight important differences in protein cargo contained in EVs when different isolation methods are used, which currently represents a major obstacle when results are compared among studies and laboratories. Nevertheless, the data shown here represents the first proteomic signature of equine uterine EVs revealing proteins with potentially interesting roles in regulating embryo/conceptus development.

### 3.1. Facing the Big Volume of the Uterine Lavage as Starting Material for EVs Recovery

Among the different factors that should be considered when selecting a method for EVs isolation from biofluids, is the volume of starting material [34]. When big volumes are obtained such as urine or from uterine lavages in our case, samples need to be concentrated prior to EVs isolation. This can be performed by ultrafiltration using different centrifugal filters or devices as the Centricon used in the present study. The use of Centricon filters offered two advantages compared to direct use of UC. Firstly, the isolation of EVs from larger sample volumes gives higher EVs yields and concentration prior to UC can avoid potential EVs loss during UC, as pointed by Heinemann et al. [35]. These authors used 150 mL of cell culture conditioned medium as starting material and obtained 81% EVs recovery using filtration compared to 23% by UC without filtration [35]. Secondly, two or three rounds of UC depending on the starting volume and very long run times of the UC can be skipped, thus providing a faster alternative to UC [35,36]. By contrast, some limitations have been pointed out, that when centrifugal filters are used, EVs can be trapped in filter pockets and therefore, it can also result in a significant loss of EV yield [37,38]. In this regard, studies comparing filters made of different materials have shown that best materials for recovering EVs from plasma, urine and EV-spiked PBS are based on regenerated cellulose membranes with pores capable of retaining particles/molecules above 10 kDa [37]. In our study, we used this type of filter but with a cut-off of 3 kDa since we also wanted to collect the free proteins present in the uterine lavages, not packed in EVs, which were also analyzed by mass spectrometry (SN samples). In our hands, the use of Centricon compared to UC (MB_UC vs. MC_CE/PBS), did not show statistically significant differences regarding the EVs size distribution, protein concentration and RNA concentration in contrast to other studies showing a reduced EVs yield with UC [38]. Although, a significantly higher number of proteins was identified using UC without concentration by ultrafiltration (MB_UC; 163.8) compared to ultrafiltration (MC_CE/PBS; 81.25), and also differences were observed in the protein cargo. Only in one comparison (ME vs. MC) a significant decrease in RNA and protein yield was obtained, which was most likely due to the stronger concentration of the samples to 500 µL in order to have a volume fitting the recommended sample volume for the Izon SEC qEV columns. To make the methods comparable, the samples of MC (CE/PBS_UC) were concentrated to the same low volume of 500 µL. In method F, the volume was only concentrated to 1 mL and PBS/trehalose used for the washing step during ultrafiltration, leading to a much better RNA and protein yield comparable to the other methods where the samples were concentrated to 3 to 4.5 mL. Depending on the pore size and filter material, loss of EVs has been reported, which could be due to the formation of precipitates in the filter (cake formation) leading to a loss of EVs [37,39].

### 3.2. Protecting the EVs during Isolation and Further Processing and Down-Stream Analysis

The aggregation of vesicles in concentrated suspensions and their deformation are factors that can affect EVs isolation and have been associated to UC [40]. Besides freezing/thawing cycles of EVs samples, isolation procedures can also induce damage of vesicles, which can impact their biological activity [41]. To reduce these effects, Bosch et al. [41] added trehalose (25 mM) to EVs isolation buffer during filtration. Trehalose is a natural and non-toxic sugar extensively used as a protein stabilizer and cryoprotectant in the food and drug industry [42,43]. In the reproductive field, it has been used in human oocyte cryopreservation, without showing a toxic effect [44]. In EVs field, trehalose has been used to minimize fusion during in vitro electroporation experiments [45] and loss of exosomes during freeze-drying (patent CN104488850A). In this line, Bosch et al., [41] showed that trehalose protects the EVs from lysis or damage during repeated free-thaw cycles, increased the number of individual particles per microgram of protein and seemed to preserve their biological activity [41]. Altogether, it made us consider using trehalose in our EVs buffer (PBS). In our study, TEM observations of EVs preparations obtained from MD_CE/TRE showed better vesicle structures and more clean preparations with less aggregates than MC_CE/PBS. Moreover, the EVs size distribution results could indicate that use of trehalose provides EVs preparations with higher amounts of 30 to 100 nm vesicles resembling exosomes and fewer very small vesicles (<30 nm), but the difference of the mean values was not significant (only three replicates). In the comparison of MD_CE/TRE vs. MC_CE/PBS, the number of identified proteins in MS analysis was significantly higher, suggesting prevention of EVs and/or protein loss during ultrafiltration. Furthermore, when trehalose was combined with ME_CE/IZON/UC, referred as MF, we observed an evident increase in both protein (1.32 ± 0.46 representing mean and SEM; to 3.69 ± 1.08 µg/µL) and RNA (0.092 ± 0.01 to 6.81 ± 2.25 ng/µL) concentrations in our preparations compared to ME_CE/IZON/UC (without trehalose), although not statistically different (*p* value = 0.056 for protein and *p* value = 0.156 for RNA).

### 3.3. Improving the Purity of Equine Uterine EVs Populations

Currently, SEC is being more and more used to isolate EVs from different types of samples including reproductive fluids [18,46,47]. By using SEC, iZON qEV columns, a population of vesicles with higher purity and integrity and with less EVs-aggregates formed during the isolation procedure has been shown [48,49]. Moreover, it has been proposed as a better method to avoid the co-isolation of non-EV components, which include soluble proteins, protein aggregates, lipoproteins (especially high- and low-density lipoproteins, HDL and LDL, respectively) [50]. With this purpose, we used iZON qEV columns in our study and performed a comparative analysis between the use of SEC and UC. The use of method ME_CE/IZON/UC seems to provide samples with less impurities and EVs aggregates and without vesicles >200 nm compared to method MC_CE/PBS. However, the EVs isolated by SEC are contained in a big volume (500 µL for 1 fraction, 1.5 mL for fractions 7–9) and have to be concentrated for EVs characterization experiments and/or down-stream omics applications. To finally concentrate the EVs fractions derived from IZON qEV SEC we used UC. The UC offered us the low cost and the capacity to obtain EVs pellet from 3 mL of pooled fractions 7–9 derived from SEC purification of 1 mL concentrated uterine lavage samples, which were resuspended in 50 µL for further analysis. Nordin et al., [38] have shown that UC can induce some EV disruption after UC for 70 min at 120,000 *g* and recommended that the recovery of intact EVs by UC should be done at a maximum speed of 100,000× *g*. In our laboratory, we have used UC protocols based on 100,000× *g* for this study, and also in previous studies to isolate oviductal and follicular EVs from different species with good results based on TEM observations [15,16,17,51,52,53]. However, considering the possibility that UC or CE processes could affect the EVs morphology, we used trehalose during the CE wash and dissolved the pellet after UC in PBS/25 mM trehalose. In addition to the prevention of EVs aggregation and cryoprotective properties [41], trehalose is also preventing protein denaturation and aggregation [42] and could thereby reduce unspecific protein contaminations in EVs samples.

Our results clearly showed that the combination of filtration by CE with a washing step with PBS/25 mM trehalose, together with SEC and finally concentration of the EVs enriched fractions by UC seemed to be the best of the tested methods for isolation of EVs from equine uterine lavages (MF). We observed that in comparison C–E, both methods MC_CE/PBS and ME_CE/IZON/UC clearly provided much lower protein (<2 µg/µL) and RNA concentrations (<0.1 ng/µL) derived from EVs compared to methods in comparisons B–C and C–D (protein 3.9–5.2 µg/µL; RNA 12–20 ng/µL) (no statistical comparison was performed among pairwise comparisons). However, when all methods were compared, a significant decrease in both protein and RNA concentrations in method ME_CE/IZON/UC was observed compared to MB_UC ± 0.01; MC_CE/Ce/PBS and MD_CE/TRE. When trehalose was also used in MF_CE/TRE/IZON/UC, we observed a tendency to increase the protein and RNA concentrations but not significant (*p* value = 0.056 for protein and *p* value = 0.156 for RNA) in comparison to ME_CE/IZON/UC. Previous comparative studies on the impact of different EV isolation methods on EV-RNA yield and purity have also shown differences among methods (e.g., kit-based EV methods with higher protein concentration versus density gradient-based isolation with higher pure EV population; [54]). As mentioned above, the main difference in the comparison of MC and ME was the concentration of the lavage samples down to 500 µL by ultrafiltration, which most likely led to a substantial loss of EVs. It has also been suggested that when a combination of methods is used as in our study, it may improve the depletion of lipoprotein and protein contaminants in EVs samples, but it can also decrease the overall yield of EVs [50]. Here, the complex method MF_CE/TRE/IZON/UC (ultrafiltration in the presence of trehalose, SEC, ultracentrifugation) revealed similar protein concentrations and only slightly lower RNA concentrations compared to the other methods without SEC. Overall, as suggested by ISEV position paper on RNA EVs [55], a compromise of yield versus purity needs to be made to avoid biasing conclusions drawn from EVs RNA and protein analysis. In our study, the use of ultrafiltration combined with SEC and UC in MF and the concentration by ultrafiltration to a limited minimal volume (1 mL) in the presence of trehalose, resulted in RNA and protein yields similar to the other, more simple methods. 

### 3.4. Avoiding Co-Isolation of Non-Vesicular RNA When Analyzing the Uterine EVs RNA Cargo

Among the different molecular cargo of EVs, the EVs RNA component has been of particular interest because of their potential use as diagnostic biomarkers, since RNA packed in EVs remains stable compared to free RNA in biofluids that can be exposed to RNases [56]. This protective effect of EVs on RNA cargo makes it possible to perform RNA analysis of frozen EV samples stored for even more than 12 years still containing RNA of high quality [57]. However, there are also stable RNAs in biofluids, which are not packed in EVs such as miRNAs, but protected by protein complexes containing AGO2 or other ribonucleoproteins [58,59] or associated with HDL and LDL particles [60]. The use of ultracentrifugation and precipitation-based strategies, such as polyethylene glycol (PEG)-based kits for EV isolation has been found to yield EVs samples that are highly contaminated with ribonucleoproteins and RNA loosely bound to EVs [61]. While EV preparations with high purity are obtained when using SEC, e.g., iZON columns, alone or combined with proteinase K and RNase A treatment. The SEC method removes protein aggregates and provides EV fractions free of protein contamination [61]. It has been demonstrated that SEC is an optimal method for EVs isolation from plasma [48,49,62]) and other types of reproductive fluids (uterine fluid [18] and seminal fluid [63]). 

Additionally, to ensure that the RNA isolated from uterine EVs RNA cargo was only confined inside the EVs and not derived from co-isolated non-vesicular RNA aggregates present in EVs preparations, a RNase protective assay was performed using EVs derived from the best method (MF). Our results showed for the EVs isolated with MF_CE/TRE/IZON/UC that no differences were found between untreated samples and samples treated with RNase, indicating that the optimized EVs isolation method MF provides RNA derived from the inside of the EVs and not from non-vesicular RNA aggregates present in EVs preparations. To assess the RNA quantity and quality of this MF-derived samples, three different RNA measurement methods were used, considering their different suitability for EVs samples [55]. The three methods (Nanodrop 3300 fluorometer, Quantus™ fluorometer, Agilent 2100 Bioanalyzer RNA 6000 Pico assay) provided similar results. Furthermore, RNA EVs profiles provided by Agilent 2100 Bioanalyzer, showed one high RNA peak representing shorter RNAs (maximum around 150 nt) and two smaller peaks representing 18S and 28S ribosomal RNAs, indicating that small RNAs are dominant in equine uterine EVs RNAs as expected and similar to other EVs RNA profiles [64,65]. Altogether, these data indicate that further experiments with these uterine EVs samples isolated by method MF can be performed without RNase treatment, it may also have unwanted effects on the EVs functionality or cargo since samples are incubated for 30 min at room temperature during RNase treatment. Moreover, additional handling of EVs preparations may also affect the EVs RNA integrity and quality, causing RNA damage, RNA fragmentation, and influence the results obtained by downstream quantitative applications.

### 3.5. Differences in EVs Protein Cargo among EVs Isolation Methods

Our results indicated differences in protein cargo among EVs isolation methods. These results are in line with other EVs studies showing different protein profiles depending on the isolation methodology [36]. Even distinct EVs protein glycosylation profiles have been obtained depending on the isolation technique [66]. Similarly, studies on EVs RNA cargo have shown that the isolation method impacts the RNA cargo as well [49,67,68,69]. Besides, Tang et al. [68] demonstrated that EVs isolated by UC, ExoQuick or Total Exosome Isolation Reagent yielded differences in miRNA sequencing analysis outcomes. For example, 588 identified miRNAs in EVs were common to all methods, while approximately 200 miRNAs were unique to each method. This represents a major obstacle for comparing studies among laboratories and highlights the importance of detailing the protocol used for isolation of EVs and protein or RNA analysis. Furthermore, this variability affects the interpretation of the obtained data with respect to potential biological functions of proteins identified in EVs.

Here, we would like to mention a few examples of proteins that were affected by the presence of trehalose during ultrafiltration (MD_CE/TRE) and the use of SEC (ME_CE/IZON/UC) or by both, since these two methods seemed to have a positive influence in the EVs isolation. Our results showed that both methods provided EVs preparations with higher purity, maintaining better morphology, and increasing the number of identified proteins in EVs, mainly when trehalose was used. For example, ADH5 was not identified by trehalose method (MD_CE/TRE) but with relatively high intensity in all three replicates of MC in the comparison ME vs. MC. ADH5 gene expression has been found in intercaruncular tissues in bovine [70] and also identified in the uterine fluid proteome of roe deer [71], where it may play a role in cellular detoxification before implantation. Besides, the use of trehalose in the EVs isolation protocol allowed or improved the identification of other proteins including PAK2, LAMB2 and SEMA4B, which may play an important role in uterine functions and embryo development. PAK2 has been shown to be essential during embryogenesis and also for adult blood vessel maintenance [72]. It plays also an important role as the regulator of cellular senescence and organismal aging [73]. LAMB2 is thought to mediate the attachment, migration and organization of cells into tissues during embryonic development by interacting with other extracellular matrix components [74]. SEMA4B gene expression has been identified to be altered throughout decidualization in murine [75], associated to mucosal remodeling and angiogenesis [76] and pointed as a negative regulator of basophil-mediated immune responses [77]. 

On the other side, the use of SEC method (ME), improved the identification of proteins such as envoplakin (EVPL), which was not detectable in almost all of the samples of the other methods and not in the supernatant. Envoplakin is highly expressed in uterine epithelial cells according to the Human Protein Atlas project database. Moreover, it has been shown that envoplakin expression decreased in pregnant porcine endometrium compared to non-pregnant, which has been associated with epithelial cell differentiation and epithelial barrier formation [78]. Coactosin-like protein (COTL1), also mainly identified when SEC was used, and has been detected in bovine uterine fluids from cyclic and pregnant animals [79]. Our results suggest that COTL1 may have been found in EVs of those animals when complete UF was analyzed. Another such protein, chloride intracellular channel 4 (CLIC4) has been recently found to be dysregulated in the endometrium of infertile women [80]. Altogether, these results show that the selection of the EVs isolation method can impact the protein cargo identified in EVs. Moreover, it can impact the detection of proteins in EVs, which have been previously identified in the uterine fluid or uterine tissues and may play key functions in the uterus as well as mediators of the uterus-embryo crosstalk via EVs. In the light of our results, the combination of using trehalose and SEC may be a good strategy to ensure the identification of proteins present in uterine EVs and are associated with important uterine functions.

Besides, we would like to briefly mention that the use of trehalose during ultrafiltration and SEC most likely reduced contaminations by serum proteins that are quite abundant in the uterine lavage samples, since the use of these methods reduced the amount of A2M and plasminogen (PLG) (in ME vs. MC), and ALB and HBB (in MD vs. MC). Similar results have been observed recently by O’Neil [18] using ultrafiltration followed by SEC to isolate EVs from ovine uterus. In the same line, isolation by SEC has resulted in higher EVs recovery and significantly less protein contamination than EVs isolated by precipitation [36,81].

### 3.6. Facing a Difficult Question: Which Is the More Appropiate Method for a Specific Fluid or Source of EVs?

In order to try to answer this question regarding the EVs from uterine lavages from the mare, we designed this study. After testing five different protocols and the combination of them, our results point to the method MF_CE/TRE/IZON/UC (combination of ultrafiltration in the presence of trehalose, SEC, ultracentrifugation) as the method provided EVs samples with more purity, without compromising the yield, i.e., RNA and protein concentration in the EVs pellet samples. In the light of our results, the advantages of using this combined method might be based on: (1) The use of trehalose during the CE washing step and the final pellet suspension resulting in less EV aggregates that can deform the EVs lead to losses during ultrafiltration and also protection of EVs structures during freezing [41]. Additionally, trehalose is also preventing protein denaturation and aggregation [42] and could thereby reduce unspecific protein contaminations in EVs samples. (2) The CE ultrafiltration allowed to reduce the big volume of starting material from 70 to 1 mL without compromising the EVs yield and also allowing to use IZON size exclusion chromatography columns afterwards; (3) The use of IZON columns led to a population of vesicles with higher purity and integrity and with less EVs aggregates. Results of mass spectrometry analysis pointed at reduced unspecific contaminations with highly abundant proteins and thereby increasing the detection of EVs-specific proteins. (4) The final use of UC to pellet the EVs samples under our conditions seems to be an efficient method to concentrate the diluted EVs samples after the IZON columns.

The results from our study in terms of differences in EVs population and purity and EVs protein cargo emphasizes the need for performing preliminary experiments to find the more appropriate isolation method adapted to the starting material or source of EVs. Based on our efforts to protect the EVs, avoiding contaminations but without compromising yields versus purity in EVs samples, we found that the method MF was the more appropriate among the five methods tested for our type of sample.

### 3.7. Exploring the uEVs Protein Components: Comparison among Species and Functions Related to Support Embryo Development in the Uterus

The comparative analysis of the uterine EVs protein cargo among species revealed overlaps in identified proteins clearly depending on the EVs isolation method. In general, different factors could be the reason for the small overlap of uterine EVs protein cargo among some of the studies: the method of EVs isolation, the method of collection of uterine fluid, the source of uterine EVs (in vivo vs. in vitro, [15]), starting material (volume uterine lavage or conditioned media), female factors (hormonal status, stress, etc.) and also species-specific differences. However, considering the EVs isolation methods used in the compared studies and even isolation with different methods from the same UF source in our study and in the ovine studies [12,18], it is clearly suggested that the EVs isolation method has a major impact on the results of EVs proteomics analysis. Besides, the comparisons in Table 2 suggest that higher similarities are obtained when the same isolation method is used despite differences in species. At this point, we also would like to mention that the use of different annotation databases and different protein identifiers represents another bottleneck when comparative analyses among species are performed. For example, the supplemental data of O’Neil et al. 2020 [18] gives RefSeq protein identifiers from mixed species, among them organisms such as Ovis aries musimon, which protein identifiers have been removed from the databases. All these points represent limitations that should be overcome to allow comparative analyses between or within species that can lead to the identification of specific but also common uterine EVs molecular cargo, which could point to functional EVs cargo and biomarkers of fertility and normal embryo development in the different species. 

Although the aim of this study was not to provide a deep analysis of the EVs protein cargo, we identified uterine EVs proteins associated with embryo support and implantation. Lipocalin P19, a progesterone induced protein, which is abundantly present in the equine uterine secretions during diestrus and early pregnancy [82,83] and with increased mRNA expression during late diestrus and Day 12 of pregnancy in luminal epithelium [84,85,86] was identified in uterine EVs in the mare. Besides, a function as the carrier of a maternal factor needed to sustain the developing embryo during pregnancy has been suggested for P19. Furthermore, P19 is incorporated into the embryonic capsule [82], being one of the most abundant proteins in the equine embryonic capsule [87,88,89], where P19 may perform some other functions involved in the maintenance of pregnancy. In the light of our results, we hypothesize that P19 might be incorporated into the equine embryo in part via uterine EVs, since it has been identified in EVs by all methods used. We cannot discard other ways of incorporation since p19 was also highly abundant in the SN preparations. 

By contrast, Furin, a protein essential for normal embryonic development [90], was identified uniquely in uterine EVs by all methods, but was not detectable in the SN samples. In humans, furin function is required for trophoblast cell invasion into the maternal endometrium [91]. Previously, differentially abundant amounts of furin were found in placenta-derived exosomes from preterm delivering pregnancies compared with at term delivering pregnancies [92].

Valosin containing protein (VCP), is another protein identified in equine uterine EVs by several methods and not detectable in SN preparations. Its expression has been localized to the cytoplasm and nucleolus of both oocytes and preimplantation embryos, and functions in meiotic arrest of mouse oocytes [93]. Moreover, it is essential for embryo development, since targeted deletion of VCP in mice results in early embryonic lethality [94]. VCP has been also associated with membrane fusion and trafficking processes in mammalian cells [95,96]. Besides, it has been suggested that VCP and tyrosine phosphorylation of this protein may have a role as a link between capacitation and the acrosome reaction [97].

## 4. Materials and Methods

### 4.1. Animals and Sample Collection

Eight warmblood mares (2 Freiberger, 6 Trotter, 4 to 18 years old) belonging to the Agrovet-Strickhof Center for Education and Research (Lindau (ZH), Switzerland) were used for sample collections from May until November 2018. All animal procedures were conducted with the permission of the veterinary inspection office of the Canton Zurich (Permission No. ZH184/17). The procedures performed had a degree of severity corresponding to grade 1.

Since the purpose of this study was to establish a protocol to examine equine uterine EVs that can be used in further studies to explore the role of these EVs in embryo-maternal interactions during the period of maternal recognition of pregnancy (MRP) in the mare, we selected a methodological procedure mimicking as much as possible a real insemination and further sample collection on day 13 after ovulation (D13). To this end, mares were examined daily for signs of estrus with a LOGIQ e ultrasound device (GE Healthcare; Glattbrugg, Switzerland). When a follicle of at least 35 mm diameter was detected in combination with uterine edema corresponding to estrus, each mare received a single dose of 1500 IU human choriogonadotropin i.v. (hCG; Chorulon; MSD Animal Health GmbH, Luzern, Switzerland); as an ovulation-inducing agent. Ultrasound checks were performed three times per day until detection of ovulation (D0) was confirmed. Then, a sham insemination was performed, by intrauterine application of INRA82 extender (4 mL). On D13, the uterine lavage of each mare was carried out by introducing 100 mL of pre-warmed phosphate buffered saline solution (PBS; #P4417-100TAB, Sigma-Aldrich Chemie GmbH, Buchs, Switzerland) into the uterine lumen. Then, a careful transrectal massage of the uterus was performed to assure an equal passage of the fluid throughout the entire uterus. Subsequently, the fluid was recovered through the tube and placed immediately on ice until serial centrifugation was performed. To induce luteolysis at the end of the experiment, 250 µg of (+)-Cloprostenol (Genestran, Graeub, Bern, Switzerland) were administered i.m. after sample collection.

### 4.2. Isolation of Equine Uterine EVs by Different Methods

After collection of uterine lavages from each mare, uterine fluid (UF) samples were transported to the laboratory on ice and rapidly processed. First, UF samples were centrifuged at 300× *g* for 15 min at 4 °C to remove the cells and blood. The supernatant was transferred to a new tube and centrifuged at 2000× *g* for 15 min at 4 °C to remove cellular debris. Then, UF samples were stored at −80 °C until EVs isolation. Subsequently, UF samples from each mare were thawed on ice and centrifuged at 12,000× *g* for 30 min at 4 °C in order to remove cellular debris, apoptotic bodies and bigger microvesicles, before EVs isolation by different methods. The pellet obtained after 12,000× *g* was suspended in PBS (#P4417-100TAB, Sigma-Aldrich Chemie GmbH) and stored for further examination and the clarified UF was used for EVs isolation.

The assessment of a total of six different EVs isolation methods (A–E) was carried out. Figure 1 shows a schematic representation of the experimental design of the study. With the first method A (MA_UC), classical ultracentrifugation (UC) 100,000× *g* for 90 min at 4 °C using fixed angle Beckman rotor (MLA-55) and Beckman bottles (No. 355,603 bottle and cap assembly, Beckman Coulter International S.A., Nyon, Switzerland) and Beckman Optima MAX-XP ultracentrifuge (Beckman Coulter International S.A.) was performed. The first UC step followed a second UC with the same parameters after washing the EVs pellet with PBS. From now on, this step is referred to as 2×. Method A (MA_UC) was used to examine if the obtained EVs concentration increased as the UF sample volume increased. Thus, in this case, the complete volume of UF of one mare (approximately 70 mL) was used to obtain EVs by MA_UC from different volumes (10 mL, 20 mL, 40 mL, and 10 mL PBS as control (no EVs expected)). The rest of the methods (B–E) were compared by two-by-two comparisons (three comparisons, Figure 1), splitting each UF sample into two samples (40 mL and 40 mL) in order to: (1) avoid individual variability of the mares or samples that could mask the results of the comparative analysis of EVs isolation methods; and (2) yield enough EVs for further EVs characterization by each method, since the use of one UF sample (70–100 mL; one mare) as starting material by all methods could limit further characterization of EVs.

In the first comparison, Methods MB_UC and MC_CE/PBS were compared two-by-two; MB_UC: UC at 100,000× *g*, 90 min, 4 °C (2×) using swinging bucket Beckman rotor MLS-50 and Beckman tubes (Ultra-clear, No.344057); MC_CE_PBS: use of Centricon filter (CE) (Centricon Plus-70 Centrifugal Filter devices, regenerated cellulose, 3kDa, 15–70 mL, Merck Millipore, Ref: UFC700308, Sigma-Aldrich Chemie GmbH) to concentrate 40 mL of UF sample to 3 to 4.5 mL according to the manufacturer’s protocol, with a washing step of the concentrate with 25 mL PBS, followed by UC of the concentrate (filled with PBS to 5ml) using the same parameters as for MB_UC.

In the second two-by-two comparison, Method C (MC_CE/PBS) was assessed with two different washing steps: (1) washing step with 25 mL PBS (MC_CE/PBS) or (2) washing step with PBS-25 mM trehalose (TRE; Sigma, T0167, Sigma-Aldrich Chemie GmbH) (MD_CE/TRE). Uterine lavage samples were concentrated (CE) to 3 to 4.5 mL.

In the third two-by-two comparison, Method C (MC_CE/PBS) was compared to a combination of CE with PBS-wash, followed by size-exclusion chromatography (SEC) using iZON_qEVs (70 original columns #1003851, Izon Science Europe Ltd, Oxford, UK) and subsequent concentration of pooled fractions 7–9 (enriched in EVs) by UC 100,000× *g*, 90 min at 4 °C (Method E, ME_CE/IZON/UC). Uterine lavage samples were concentrated (CE) to 500 µL. When iZON_qEVs columns were used, all fractions from 1 to 20 were collected (0.5 mL/fraction) according to manufacturer´s instructions. An aliquot of all fractions (1–20) was used to analyze the efficacy of SEC iZON qEVs to separate EVs from soluble proteins in UF by examining the protein concentration of the collected fractions (Supplementary Figure S4).

A total of four different replicates for each method were performed using 16 different uterine lavages from eight mares. All resulting pellets from each EVs isolation method were diluted in 50 µL of PBS and aliquots were stored for subsequent characterization experiments and analysis of the EVs protein content by mass spectrometry.

Finally, based on the EVs yield, purity, and protein cargo profiles, a sixth last method (MF) was tested by combining the methods deriving the best results. This method was based on method ME_CE/IZON/UC with some minor modifications according to the optimization steps observed in the other methods: (1) UF was concentrated only to 1 mL with Centricon to avoid that EVs become trapped in the filter pores; (2) a washing step with PBS/25 mM trehalose instead of PBS only was integrated to prevent aggregation of proteins and vesicles due to protein denaturation; (3) only one UC was performed to concentrate samples after SEC instead of two to reduce loss of EVs; and (4) the obtained EVs pellets by MF were diluted in 50 µL of PBS with trehalose (25 mM) to protect EVs and prevent EVs aggregation during storage and further sample processing.

### 4.3. Characterization of Equine Uterine EVs by Transmission Electron Microscopy (TEM) and Analysis of EVs Size Distribution

For TEM observations and evaluation of EVs size distribution, EVs suspensions were diluted in PBS and fixed in glutaraldehyde (freshly prepared) (1% final concentration). Then, three microliters of each EVs sample were placed on the formvar carbon-coated grid for 5 min and washed with distilled water (three times). For negative contrast, the samples were incubated in 2% water solution of uranyl acetate (30 s three times, 5 μL) and left to dry in the small drop (near 1 μL) of last solution. The micrographs were obtained using TEM HITACHI HT 7700 Elexience at 80 kV (with a charge-coupled device camera AMT) and JEM 1011 (Jeol Ltd., Tokyo, Japan) equipped with a Gatan digital camera driven by Digital Micrograph software (Gatan Inc., Pleasanton, CA, USA) at 100 kV.

The processing of the photos and vesicle size calculation were carried out by ImageJ software. For TEM analysis, three different replicates of EVs samples from the four different methods were analyzed and more than 200 vesicles were counted/sample.

For immunogold labelling of CD9 exosomal marker in EVs preparations and TEM observations, EVs suspensions were diluted in PBS and fixed in Paraformaldehyde 8% solution in a concentration (concentration 1:2). Then, EVs samples were fixed in 4% formaldehyde and diluted with PBS (10 μL of sample + 10 μL of PBS). Then, 2 μL of diluted samples were applied to the surface of a nickel grids covered with a formvar/carbon film and incubated in a humid chamber for 15 min. The grids were washed with PBS (3 times for 10 s) and transferred for 30 min onto drops of PBS with 3% BSA in a humid chamber. Then the grids with the samples were transferred to the drops of the first antibodies (mouse anti-human CD9, Bio-Rad Laboratories AG, Cressier, Switzerland) diluted in PBS with 1% BSA for 60 min in a humid chamber. In parallel, some of the grids were transferred to PBS drops with 1% BSA as a negative control. Then the samples were washed from the first antibodies by incubation on PBS drops with 1% BSA (six times for 5 min). Then, the grids with the samples were transferred to the drops of the second antibodies (goat anti-mouse IgG conjugated with 10 nm gold particles) diluted in PBS with 1% BSA for 60 min in a humid chamber. Then, the samples were washed from the second antibodies by incubation on drops of PBS with 1% BSA (three times for 5 min) and PBS (three times for 5 min). The samples were additionally fixed for 5 min with 1% glutaraldehyde on PBS on drops in a humid chamber. The grids were washed with distilled water (three times for 20 s) and contrasted with a 2% aqueous solution of uranyl acetate (three times for 20 s). The last drop of uranyl acetate was removed with filter paper and the samples were air dried at room temperature. Micrographs were obtained using a JEM1011 TEM (Jeol Ltd., Japan) equipped with a Gatan digital camera driven by Digital Micrograph software (Gatan Inc.) at 100 kV.

### 4.4. Protein Profile of Equine Uterine EVs

#### 4.4.1. Protein Quantification

Measurements of protein concentration in all MVs and EVs preparations were obtained after 12,000× *g* and 100,000× *g* centrifugations, respectively, and fractions derived from SEC iZON_qEVs, were performed using the Pierce™ BCA Protein Assay (Pierce™ BCA Protein Assay Kit, Thermo Fisher/Life Technologies Europe BV, Zug, Switzerland), according to the manufacturer’s instructions.

#### 4.4.2. SDS-PAGE

To examine differences in the protein profile between MVs and EVs preparations, both types of samples were separated by gradient Sodium Dodecyl Sulphate-Polyacrylamide gel electrophoresis (SDS-PAGE) according to Laemmli [98], in a 4 to 20% polyacrylamide gel (Stain-free gel, #4568093, Bio-Rad Laboratories AG). After SDS-PAGE, proteins profiles were visualized by ChemiDoc MP Imaging System (Stain free blots, Bio-Rad Laboratories AG).

#### 4.4.3. Western Blotting

To characterize the equine uterine EVs with known exosomal markers (Table 1) by Western blotting, proteins from MVs and EVs preparations were trans-blotted to nitrocellulose protean membranes (Trans-Blot Turbo Transfer Mini Nitrocel. membrane, #170-4158, Bio-Rad Laboratories AG) with a Trans-Blot Turbo Transfer System (Bio-Rad Laboratories AG, program mixed, 7 min, transfer). The transfer was followed by 1 h membrane incubation with blocking solution of 5% skim milk (Sigma 70166) in TBS-Tween 0.05% (TBS #1,706,435, Bio-Rad Laboratories AG; Tween 20; #P9416, Fisher Scientific AG, Reinach, Switzerland) (TBS-T). Incubation of membranes with primary antibodies diluted in blocking solution (TBS-T milk 5%) was performed overnight at 4 °C. Then, the membranes were washed with TBS-T three times, 10 min each, before the incubation with secondary antibodies were diluted in TBS-T for 1 h at room temperature. Antibodies and dilutions used for Western Blotting experiments are detailed in Table 1. Subsequently, the membranes were washed with TBS-T three times, 15 min each, before developing the immune blot with the Clarity Max Western Blotting ECL Substrates (#170-5062, Bio-Rad Laboratories AG). ChemiDoc MP Imaging System was used to detect proteins after Western blot (Bio-Rad Laboratories AG).

#### 4.4.4. Analysis of Equine Uterine EVs Protein Content by Mass Spectrometry

To prepare samples for mass spectrometry, 50 mM dithiotreitol (DTT)/50 mM NH4HCO3 was added to give a final concentration of 5 mM and incubated for 30 min at 37 °C for protein reduction. Cysteines were alkylated with iodoacetamide (final concentration 15 mM) for 30 min in the dark. For protein digestion Lys-C (Wako Chemicals) was added (enzyme/substrate ratio 1/100) and incubated for 4 h at 37 °C. Then, a second overnight digestion step with sequencing grade modified porcine trypsin (enzyme/substrate ratio of 1/50) at 37 °C was performed. LC-MS/MS analysis was done with an Ultimate 3000 nano-LC system (Thermo Fisher Scientific, Erlangen, Germany) coupled to a QExactive HF-X mass spectrometer (Thermo Fisher Scientific). Peptides were injected at a flow rate of 20 µL/min to a trap column (Acclaim^®^ PepMap 100, 100 μm × 2 cm, nanoViper C18, 5 μm, 100 Å, Thermo Scientific) and separated at a flow rate of 200 to 250 nl/min using EasySpray columns (PepMap RSLC C18, 75 µm ID, 2 µm, Thermo Fisher Scientific) and 0.1% formic acid as solvent A. The chromatography method consisted of two consecutive gradients from 3% to 25% solvent B (0.1% formic acid in acetonitrile) in 30 min and from 25% to 40% B in 5 min. For data dependent acquisition cycles of one full scan (350 to 1600 m/z) at a resolution of 60k and up to 12 data-dependent MS/MS scans at a resolution of 15k were used. Thermo RAW files were analyzed using MaxQuant (v. 1.6.1.0) [99] and the equine subset of the NCBI RefSeq protein database. For protein identification, a false discovery date (FDR) < 0.01 at the peptide and protein level was applied.

Analysis of mass spectrometry data was based on normalized and log2-transformed mass spectrometry intensities. Then, lists with proteins enriched in EVs or in the supernatant after ultracentrifugation (SN), i.e., free proteins in the uterine lavage, were prepared for functional enrichment analysis. Proteins only detectable in EVs (all methods, no. of replicates ≥2) and not in SN and proteins with a log2 fold change of ≥2 between EVs samples and SN samples were used as a list of proteins enriched in EVs. Likewise, proteins only detectable in SN (in both samples) or with a log2 fold change (SN vs. EVs) of ≥2 were used as a list of proteins enriched in SN.

Additionally, pairwise quantitative analyses were performed among method comparisons. For this, corresponding LFQ values were loaded in Perseus V1.5.3.2, filtered for at least two values in at least one group. To handle missing values, the Perseus imputation feature was used. *p*-values were calculated by a paired two-sample t-test.

MS data have been deposited to the ProteomeXchange Consortium via PRIDE [100,101] partner repository with the dataset identifier PXD022264.

### 4.5. Data Mining and Bioinformatics Analysis of EVs Content

Gene symbols and NCBI Entrez Gene IDs (mare and putative human orthologs) were mapped for all proteins identified using bioinformatics custom tools integrated in a local Galaxy installation. Custom database tools (NCBI annotation mapper, Mammalian Annotation database, MAdb) [102] were used to assign known or putative human orthologous genes. Human gene identifiers or symbols were used for subsequent functional annotation.

To obtain information about overrepresented biological functions and pathways for the protein sets obtained by the different EVs isolation methods, the Metascape tool was used (https://metascape.org/gp/index.html#/main/step1) [103]. Besides, to compare the biological functions of proteins highly enriched in EVs obtained by most of the isolation methods, or only specific methods or only in the supernatant (SN), Metascape tool “membership analysis” and “enrichment analyses were used. To represent comparisons among proteins categorized with different membership by Metascale tool, Jvenn, an integrative tool for comparing lists with Venn diagrams, was used http://jvenn.toulouse.inra.fr/app/example.html [104].

### 4.6. Analyzing RNA Quality Profile of Equine Uterine EVs

#### 4.6.1. RNA Isolation and RNA Quantification

To extract RNA from EVs isolated by different methods, QIAzol lysis reagent (QIAGEN AG, Hombrechtikon, Switzerland) followed by miRNeasy micro kit (QIAGEN AG) was used according to the manufacturer´s instructions.

Considering that RNA concentration can vary among RNA quantification methods and even more when low concentrations are measured [105], different RNA quantification methods were used in our study: (1) Agilent RNA 6000 Pico assay (Agilent 2100 Bioanalyzer, Agilent Technologies Schweiz AG, Basel, Switzerland) for RNA quantity and quality profiles of EVs samples; (2) Nanodrop 3300 (Witec AG, Sursee, Switzerland) with RiboGreen Assay for RNA quantification (Thermo Fisher/Life Technologies Europe BV) and (3) Quantus™ Fluorometer (Promega AG, Dübendorf, Switzerland) together with QuantiFluor RNA System kit (Promega AG).

#### 4.6.2. RNase Protection Assay

To determine if the RNA isolated from EVs preparations was only confined within EVs and not derived from impurities of non-vesicular RNA aggregates present in EVs preparations, a RNase protective assay was performed. For this assay, EVs obtained by Method F were used. Isolated EVs were incubated with RNase A (RNase A, DNase-, and protease-free, ref EN0531, Thermo Fisher Scientific, final concentration 0.1 mg/mL) for 30 min at room temperature to degrade unprotected RNA. Additionally, in parallel, other aliquots of the same EVs samples were treated with RNase A (0.1 mg/mL) and 1% Triton X-100 (Sigma-Aldrich Chemie GmbH) (degraded RNA control). Treatment with Triton X-100 enhances membrane disruption and promotes RNA release, which is degraded by RNase A. Besides, other aliquots of the same samples remained untreated as controls (intact RNA). After all the three treatments (RNase/Triton +/+; +/−; −/−), EVs samples were directly transfer to RNA isolation buffer (Qiazol, QIAGEN AG) and the RNA isolation protocol was performed. Subsequently, concentration and quality of extracted RNA samples from different treatments were determined by different quantification methods.

## 5. Conclusions

Our study showed that the combination of ultrafiltration with addition of trehalose, size exclusion chromatography, and ultracentrifugation is an optimal strategy to isolate equine uterine EVs with good yield and purity from low-volume uterine lavage samples. The obtained proteomic data derived from the samples isolated by the tested protocols provided the first proteomic signature of equine uterine EVs in cyclic mares and revealed proteins with potential key roles in regulating embryo/conceptus development. The established optimized protocol can be applied in future studies to determine the role of uterine EVs during MRP in the mare. Besides, our study emphasizes the need for performing preliminary experiments to find the best EVs isolation method based on type of sample, amount of starting material together with the downstream application, and in order to obtain a population of EVs with high purity and sufficient yield. Our results highlight important differences in EVs protein cargo among isolation methods, which currently represents a major obstacle when results are compared among studies and laboratories. This calls for caution with respect to data interpretation and underlining the importance of detailing the protocol used for EVs isolation and further processing.

## Figures and Tables

**Table 1:** Overlap of percentage of identified proteins in uterine EVs among studies in different or the same species and using different or similar EVs isolation method. UF: uterine fluid; CE: Centricon ultrafiltration device; IZON: size exclusion chromatography; UC: ultracentrifugation.

EVs Source/% Overlap	Equine UF	Ovine UF_2	Ovine UF_1	Bovine UF	Human UF	EVs Isolation Method	Reference
Equine UF (1030 proteins)		48	2	11	31	Combination of CE_3kDa/IZON/UC concentration	Almiñana et al. 2020
Ovine UF_2 (1332)	37		3	12	33	Combination of CE_100kDa/IZON/ultrafiltration device concentration	O´neil et al., 2020 [1]
Ovine UF_1 (192)	13	21		11	13	Precipitation solution (Exo-quick-tc)	Burns et al., 2014 [2]
Bovine UF (596)	19	28	4		22	Precipitation solution (Exo-quick-tc)	Kusama et al., 2018 [3]
Human UF (964)	33	45	3	14		Combination UC/optiprep density gradient	Greening et al., 2016 [4]

**Table 2:** Antibodies and dilutions used for Western Blotting experiments.

Marker	Primary Antibody Reference	Dilution	Secondary Antibody Reference	Dilution
CD9	Anti-CD9 Mouse Monoclonal Antibody, Clone MM2/57, MCA469GT, Bio-Rad	1:500	anti-mouse m-IgGκ BP-HRP Santa Cruz sc-516102	1:5000
CD81	Anti-CD81 Rabbit Monoclonal Antibody abbexxa, abx242949	1:1000	goat anti-rabbit IgG-HRP Santa Cruz sc-2004	1:8000
TSG101	Anti-TSG101 Rabbit Polyclonal Antibody, PA5-31260 Invitrogen, Thermofisher	1:1000	goat anti-rabbit IgG-HRP Santa Cruz sc-2004	1:8000
ALIX	Anti-ALIX Mouse Monoclonal Antibody Santa Cruz sc-53540	1:500	anti-mouse m-IgGκ BP-HRP Santa Cruz sc-516102	1:10,000
FLOT1	Anti-Flotilllin-1 Mouse Monoclonal Antibody Santa Cruz sc-74566	1:500	anti-mouse m-IgGκ BP-HRP Santa Cruz sc-516102	1:5000
ANXA1	Anti-ANX-I Mouse Monoclonal Antibody Santa Cruz sc-12740	1:500	anti-mouse m-IgGκ BP-HRP Santa Cruz sc-516102	1:10,000
ANXA2	Anti-ANX-II Mouse Monoclonal Antibody Santa Cruz sc-28385	1:500	anti-mouse m-IgGκ BP-HRP Santa Cruz sc-516102	1:10,000
HSP70	Anti-HSP70 Mouse Monoclonal Antibody Santa Cruz sc-66048	1:500	anti-mouse m-IgGκ BP-HRP Santa Cruz sc-516102	1:10,000
HLA-A	Anti-HLA-A Mouse Monoclonal Antibody Santa Cruz sc-390473	1:500	anti-mouse m-IgGκ BP-HRP Santa Cruz sc-516102	1:10,000
APOA1	Anti-APOA1 Mouse Monoclonal Antibody Santa Cruz sc-58230	1:500	anti-mouse m-IgGκ BP-HRP Santa Cruz sc-516102	1:10,000

## Data Availability

The data that support the findings of this study are available from the corresponding author upon reasonable request.

## Supplementary Materials

The following are available online at https://www.mdpi.com/1422-0067/22/2/979/s1.
