# Supplementary material for: Isolation and Characterization of Equine Uterine Extracellular Vesicles: A Comparative Methodological Study
Source: Int J Mol Sci. 2021 Jan 19;22(2):979. doi: 10.3390/ijms22020979 (PMC7835857; doi:10.3390/ijms22020979)
Supplement: Supplementary file 1 [file ijms-22-00979-s001.zip › Figure_S2.pdf]

Gene ID	Gene Symbol	Description	Hsa gene ID	Hsa symbol	Description	RefSeq Protein Accession	MC_CE/ PBS_R1	MC_CE/ PBS_R2	MC_CE/ PBS_R3	MC_CE/ PBS_R4	MD_CE/ Tre_R1	MD_CE/ Tre_R2	MD_CE/ Tre_R3	MD_CE/ Tre_R4	MC_CE/ PBS_SN _R1	MD_CE/ Tre_SN_ R1	ME_CE/ Izon/UC _R2	ME_CE/ Izon/UC _R3	ME_CE/ Izon/UC _R4	MC_CE/ PBS_R2	MC_CE/ PBS_R3	MC_CE/ PBS_R4
100061692	A2M	alpha-2-macroglobulin	2	A2M	alpha-2-macroglobulin	XP_001499173	2.00	2.00	2.00	6.67	2.00	6.83	7.24	8.33	8.10	8.43	2.00	2.00	8.87	8.18	2.00	11.38
100068225	VCP	valosin containing protein	7415	VCP	valosin containing protein	XP_023485358	10.52	11.81	10.07	10.50	9.86	10.70	9.58	10.30	2.00	2.00	11.19	10.77	10.95	12.51	12.45	10.88
100053810	MMP26	matrix metallopeptidase 26	56547	MMP26	matrix metallopeptidase 26	XP_001503575	7.34	7.31	7.62	5.30	7.12	6.26	6.29	2.00	2.00	6.30	2.00	8.05	7.59	2.00	9.42	8.05
100054109	HBB	hemoglobin, beta	3043	HBB	hemoglobin subunit beta	NP_001157490	6.79	6.70	2.00	4.36	6.94	7.75	3.92	5.04	8.97	11.95	8.93	8.25	10.23	8.37	9.97	10.47
100053541	MME	membrane metalloendopeptidase	4311	MME	membrane metalloendopeptidase	XP_023476917,XP_	10.39	10.57	10.39	8.71	10.50	9.54	9.63	8.17	7.02	7.60	11.31	11.59	12.82	10.38	11.96	11.85
100067672	VNN2	vanin 2	8875	VNN2	vanin 2	XP_023506881	8.30	2.00	5.65	2.00	9.45	5.68	6.83	2.00	9.38	8.89	11.41	2.00	10.50	9.02	8.84	10.67
100054071	STC1	stanniocalcin 1	6781	STC1	stanniocalcin 1	XP_001493245	7.45	5.52	6.34	2.00	7.71	7.01	6.98	2.00	10.33	9.76	2.00	2.00	2.00	2.00	8.45	8.80
111772846	LOC111772846	alcohol dehydrogenase class-3	128	ADH5	alcohol dehydrogenase 5 (class II	XP_023493440	7.94	2.00	7.71	2.00	7.94	8.21	2.00	7.80	2.00	2.00	2.00	2.00	2.00	9.14	10.31	2.00
100058677	CYSRT1	cysteine rich tail 1	375791	CYSRT1	cysteine rich tail 1	XP_023485229	2.00	2.00	2.00	2.00	8.46	7.26	2.00	2.00	2.00	2.00	9.86	10.00	2.00	2.00	2.00	2.00
Gene ID	Gene Symbol	Description	Hsa gene ID	Hsa symbol	Description	RefSeq Protein Accession	MC_CE/ PBS_R1	MC_CE/ PBS_R2	MC_CE/ PBS_R3	MC_CE/ PBS_R4	MD_CE/ Tre_R1	MD_CE/ Tre_R2	MD_CE/ Tre_R3	MD_CE/ Tre_R4	MC_CE/ PBS_SN _R1	MD_CE/ Tre_SN_ R1	ME_CE/ Izon/UC _R2	ME_CE/ Izon/UC _R3	ME_CE/ Izon/UC _R4	MC_CE/ PBS_R2	MC_CE/ PBS_R3	MC_CE/ PBS_R4
100034189	FN1	fibronectin 1	2335	FN1	fibronectin 1	XP_023498053,XP_	9.49	10.26	11.08	9.09	8.50	10.08	10.69	9.41	7.51	7.78	8.80	11.43	10.79	11.79	12.10	12.52
100034213	P19	P19 lipocalin	3934	LCN2	lipocalin 2	NP_001075978	9.82	10.03	10.69	8.87	9.88	10.24	10.58	10.17	12.54	12.35	2.00	9.70	8.23	2.00	10.66	10.30
100062154	MTHFD1	methylenetetrahydrofolate dehydrogenase, cy	4522	MTHFD1	methylenetetrahydrofolate dehydr	XP_001499181	2.00	6.24	5.78	7.54	6.84	7.34	6.18	7.36	2.00	2.00	8.53	7.81	8.58	9.72	8.60	2.00
100629603	JCHAIN	joining chain of multimeric IgA and IgM	3512	JCHAIN	joining chain of multimeric IgA an	NP_001271464	10.58	11.20	11.16	11.37	10.77	10.78	11.27	11.26	8.75	8.82	13.43	12.79	13.04	13.59	13.93	14.34
100064107	CALR	calreticulin	811	CALR	calreticulin	XP_001504932	9.50	9.03	8.66	8.39	9.51	9.05	9.72	8.41	5.46	5.89	10.69	9.99	10.30	11.56	10.46	10.58
100033897	GAPDH	glyceraldehyde-3-phosphate dehydrogenase	2597	GAPDH	glyceraldehyde-3-phosphate dehy	NP_001157328	9.88	10.41	9.68	10.57	9.67	9.94	8.90	10.28	8.07	8.12	11.43	11.33	11.36	11.81	12.44	11.30
100068931	FURIN	furin, paired basic amino acid cleaving enzym	5045	FURIN	furin, paired basic amino acid clea	XP_023505390	10.29	10.39	9.48	9.09	10.01	10.10	9.23	8.77	2.00	2.00	11.46	12.72	12.96	10.81	12.85	11.78
100033837	HSP90AA1	heat shock protein 90 alpha family class A m	3320	HSP90AA1	heat shock protein 90 alpha famil	XP_023483460	2.00	2.00	2.00	2.00	2.00	2.00	2.00	2.00	6.49	6.99	9.35	9.93	2.00	9.21	8.74	2.00
100034206	ALB	albumin	213	ALB	albumin	NP_001075972	7.15	7.46	7.45	7.96	6.59	7.83	7.44	7.62	12.80	12.95	10.86	9.41	12.74	10.62	8.71	11.33
100055819	ECM2	extracellular matrix protein 2	1842	ECM2	extracellular matrix protein 2	XP_023483400	10.65	10.78	9.70	9.53	10.52	11.61	10.33	9.01	8.60	8.48	11.55	12.15	11.07	10.53	11.27	10.59
100058853	CLIC1	chloride intracellular channel 1	1192	CLIC1	chloride intracellular channel 1	XP_014589790	11.27	11.05	11.34	10.47	10.77	11.21	11.23	10.24	9.50	8.81	13.19	13.19	14.10	12.60	12.36	12.72
100070095	TPP1	tripeptidyl peptidase 1	1200	TPP1	tripeptidyl peptidase 1	XP_023501673	8.13	2.00	7.42	7.23	8.11	8.18	7.27	7.28	5.79	5.55	10.17	10.79	10.18	9.06	9.93	8.89
100066144	B4GALT3	beta-1,4-galactosyltransferase 3	8703	B4GALT3	beta-1,4-galactosyltransferase 3	XP_023496606	9.70	10.10	9.07	7.52	9.27	9.42	9.06	6.43	2.00	2.00	10.22	11.26	9.95	2.00	10.86	8.03
111772887	LOC111772887	deleted in malignant brain tumors 1 protein-lik	1755	DMBT1	deleted in malignant brain tumors	XP_023493734	2.00	8.04	7.07	8.60	2.00	7.87	7.41	8.91	5.61	2.00	11.38	12.81	10.66	9.23	12.22	9.31
100147201	EPS8L1	EPS8 like 1	54869	EPS8L1	EPS8 like 1	XP_023506210	6.63	6.32	6.57	2.00	6.98	6.05	6.99	2.00	2.00	2.00	10.31	10.09	9.88	8.85	8.76	2.00
100054320	ANXA2	annexin A2	302	ANXA2	annexin A2	XP_023492402	2.00	2.00	2.00	2.00	2.00	2.00	2.00	2.00	4.90	4.95	11.45	11.01	2.00	9.90	8.35	2.00
100058503	F5	coagulation factor V	2153	F5	coagulation factor V	XP_023496285	7.32	7.03	7.13	7.07	7.45	6.97	6.71	2.00	6.70	6.50	2.00	10.01	10.93	2.00	7.63	8.10
100050531	PLG	plasminogen	5340	PLG	plasminogen	XP_014593633	2.00	2.00	9.12	7.03	2.00	7.42	8.65	6.32	2.00	2.00	2.00	2.00	8.14	9.82	2.00	10.03
100068724	IQGAP1	IQ motif containing GTPase activating proteir	8826	IQGAP1	IQ motif containing GTPase activ	XP_023505306,XP_	9.33	8.30	8.95	9.19	8.42	9.57	8.66	8.62	6.58	2.00	11.05	10.06	11.53	2.00	2.00	9.37
102147792	MUC5B	mucin 5B, oligomeric mucus/gel-forming	727897	MUC5B	mucin 5B, oligomeric mucus/gel-f	XP_023510736	2.00	6.46	2.00	6.61	2.00	6.46	2.00	6.53	2.00	2.00	9.91	12.53	2.00	2.00	9.44	2.00
100054482	MYH9	myosin heavy chain 9	4627	MYH9	myosin heavy chain 9	XP_023487015	6.40	5.27	6.11	8.02	6.35	6.79	2.00	8.00	2.00	2.00	9.03	8.62	8.23	2.00	2.00	2.00
100066142	SDF4	stromal cell derived factor 4	51150	SDF4	stromal cell derived factor 4	XP_023491823	2.00	4.32	2.00	2.00	3.33	2.00	2.00	2.00	2.00	2.00	10.08	10.67	10.72	2.00	10.44	2.00
100068209	B4GALT1	beta-1,4-galactosyltransferase 1	2683	B4GALT1	beta-1,4-galactosyltransferase 1	XP_023483236	2.00	2.00	6.21	2.00	5.53	4.84	5.59	2.00	2.00	2.00	9.00	9.97	2.00	6.79	2.00	2.00
100052421	PRDX1	peroxiredoxin 1	5052	PRDX1	peroxiredoxin 1	XP_005607147	2.00	2.00	2.00	7.34	2.00	7.15	2.00	8.56	6.75	7.24	10.20	10.17	2.00	9.11	2.00	2.00
100066002	UNC5CL	unc-5 family C-terminal like	222643	UNC5CL	unc-5 family C-terminal like	XP_023480664	2.00	5.89	6.40	6.45	2.00	5.65	2.00	2.00	2.00	2.00	9.01	8.26	9.42	2.00	2.00	7.83
100071457	CLIC4	chloride intracellular channel 4	25932	CLIC4	chloride intracellular channel 4	XP_023491248	2.00	2.00	2.00	2.00	2.00	2.00	2.00	2.00	2.00	2.00	7.95	7.79	2.00	2.00	2.00	6.49
100053824	SLC6A14	solute carrier family 6 member 14	11254	SLC6A14	solute carrier family 6 member 14	XP_001488209	2.00	2.00	2.00	2.00	2.00	2.00	2.00	2.00	2.00	2.00	6.36	7.14	2.00	2.00	2.00	2.00
100072548	CTNNA1	catenin alpha 1	1495	CTNNA1	catenin alpha 1	XP_023473380	2.00	2.00	7.09	7.97	2.00	6.91	2.00	6.83	2.00	2.00	8.76	2.00	2.00	2.00	2.00	2.00
100070195	COTL1	coactosin like F-actin binding protein 1	23406	COTL1	coactosin like F-actin binding prot	XP_001499912	2.00	2.00	2.00	2.00	2.00	2.00	2.00	2.00	2.00	4.93	8.86	9.16	10.08	2.00	2.00	2.00
100051013	TUBB	tubulin beta class I	203068	TUBB	tubulin beta class I	XP_001491228	2.00	2.00	2.00	7.44	2.00	2.00	2.00	2.00	2.00	2.00	10.11	9.46	9.23	2.00	2.00	2.00
100051395	EVPL	envoplakin	2125	EVPL	envoplakin	XP_023507756	2.00	2.00	2.00	2.00	2.00	2.00	2.00	2.00	2.00	2.00	9.26	8.21	2.00	2.00	2.00	2.00
100064004	TBC1D10A	TBC1 domain family member 10A	83874	TBC1D10A	TBC1 domain family member 10A	XP_023502630,XP_	2.00	2.00	2.00	2.00	2.00	2.00	2.00	2.00	2.00	2.00	4.38	2.00	7.61	2.00	2.00	2.00
100050482	RNH1	ribonuclease/angiogenin inhibitor 1	6050	RNH1	ribonuclease/angiogenin inhibitor '	XP_023510598	2.00	2.00	2.00	2.00	2.00	3.81	2.00	2.00	2.00	2.00	6.79	2.00	7.63	2.00	2.00	2.00
Gene ID	Gene Symbol	Description	Hsa gene ID	Hsa symbol	Description	RefSeq Protein Accession	MC_CE/ PBS_R1	MC_CE/ PBS_R2	MC_CE/ PBS_R3	MC_CE/ PBS_R4	MD_CE/ Tre_R1	MD_CE/ Tre_R2	MD_CE/ Tre_R3	MD_CE/ Tre_R4	MC_CE/ PBS_SN _R1	MD_CE/ Tre_SN_ R1	ME_CE/ Izon/UC _R2	ME_CE/ Izon/UC _R3	ME_CE/ Izon/UC _R4	MC_CE/ PBS_R2	MC_CE/ PBS_R3	MC_CE/ PBS_R4
100067589	LOC100067589	N-acetyllactosaminide alpha-1,3-galactosyltra	14594	Ggta1	glycoprotein galactosyltransferase	XP_023484558	9.52	7.28	8.65	6.70	8.57	2.00	8.60	6.02	2.00	2.00	2.00	2.00	9.90	2.00	8.96	9.92
100051840	TUBA1A	tubulin alpha 1a	7846	TUBA1A	tubulin alpha 1a	XP_001504224	6.08	6.90	2.00	2.00	6.20	6.84	5.79	7.16	5.79	6.56	2.00	2.00	2.00	2.00	2.00	2.00
100062583	APOA1	apolipoprotein A1	335	APOA1	apolipoprotein A1	XP_005611649	6.51	6.64	6.52	2.00	7.47	7.15	6.93	2.00	8.67	8.93	2.00	2.00	8.69	2.00	2.00	9.39
100052735	HNRNPK	heterogeneous nuclear ribonucleoprotein K	3190	HNRNPK	heterogeneous nuclear ribonucleo	XP_003364038	2.00	7.23	2.00	7.82	2.00	8.52	7.63	8.07	2.00	2.00	2.00	9.11	2.00	9.37	9.26	2.00
100070060	MUC4	mucin-4	4585	MUC4	mucin 4, cell surface associated	XP_023479343,XP_	6.06	8.07	7.74	8.89	6.53	8.69	9.35	9.38	2.00	2.00	10.77	12.41	9.55	10.87	11.77	9.84
102150140	SMIM22	small integral membrane protein 22	440335	SMIM22	small integral membrane protein 2	XP_005599043	2.00	5.94	2.00	5.68	2.00	6.75	5.59	6.54	2.00	2.00	7.84	9.05	2.00	7.52	8.87	2.00
100462689	SERPINA14	serine peptidase inhibitor clade A (alpha-1 ant	12	SERPINA3	serpin family A member 3	XP_014591083	8.55	7.27	10.04	7.71	9.25	9.41	9.99	8.79	6.38	6.78	2.00	9.63	11.17	8.07	10.77	10.49
100629766	MUC16	mucin 16, cell surface associated	94025	MUC16	mucin 16, cell surface associated	XP_023501276	6.16	2.00	2.00	2.00	6.34	6.24	6.28	2.00	2.00	2.00	8.47	8.77	8.98	2.00	8.50	8.97
100054410	DPYSL2	dihydropyrimidinase like 2	1808	DPYSL2	dihydropyrimidinase like 2	XP_003364519	2.00	2.00	2.00	2.00	2.00	7.20	2.00	6.95	2.00	2.00	8.63	8.56	2.00	8.41	8.44	2.00
100051383	TOLLIP	toll interacting protein	54472	TOLLIP	toll interacting protein	XP_023510580	2.00	2.00	2.00	2.00	5.55	6.33	2.00	2.00	2.00	2.00	2.00	2.00	6.87	2.00	7.63	2.00
100062752	CALM1	calmodulin 1	801	CALM1	calmodulin 1	XP_023484032	2.00	2.00	2.00	2.00	5.39	5.95	6.27	6.00	2.00	2.00	2.00	2.00	7.02	2.00	6.86	7.89
100066008	TPPP3	tubulin polymerization promoting protein famil	51673	TPPP3	tubulin polymerization promoting p	XP_005608387	2.00	2.00	2.00	6.21	5.07	7.06	2.00	7.44	2.00	2.00	2.00	7.78	6.75	7.26	9.23	2.00
100065490	DYNC1LI2	dynein cytoplasmic 1 light intermediate chain	1783	DYNC1LI2	dynein cytoplasmic 1 light interme	XP_023492902	2.00	2.00	2.00	9.83	2.00	8.68	6.86	9.13	2.00	2.00	2.00	2.00	2.00	6.87	2.00	2.00
100049942	ARHGDIA	Rho GDP dissociation inhibitor alpha	396	ARHGDIA	Rho GDP dissociation inhibitor alp	XP_023507541	2.00	2.00	2.00	2.00	7.78	2.00	2.00	6.63	6.60	5.91	9.17	2.00	2.00	2.00	9.56	9.65
100051132	ATP6V1G1	ATPase H+ transporting V1 subunit G1	9550	ATP6V1G1	ATPase H+ transporting V1 subur	XP_023484509	2.00	2.00	4.19	2.00	5.38	4.10	5.11	4.79	2.00	2.00	2.00	2.00	2.00	2.00	2.00	2.00
100052695	SPTBN1	spectrin beta, non-erythrocytic 1	6711	SPTBN1	spectrin beta, non-erythrocytic 1	XP_005600040	2.00	2.00	2.00	7.36	6.73	6.25	2.00	7.31	2.00	2.00	9.68	2.00	2.00	2.00	2.00	2.00
100056061	S100A12	S100 calcium binding protein A12	6283	S100A12	S100 calcium binding protein A12	XP_001494448	2.00	2.00	2.00	6.85	2.00	6.92	2.00	8.02	2.00	2.00	2.00	2.00	2.00	2.00	2.00	5.69
100050188	GGT1	gamma-glutamyltransferase 1	2678	GGT1	gamma-glutamyltransferase 1	XP_005612420,XP_	2.00	2.00	2.00	2.00	2.00	5.67	2.00	5.83	2.00	2.00	2.00	2.00	2.00	2.00	2.00	2.00
100060183	PAK2	p21 (RAC1) activated kinase 2	5062	PAK2	p21 (RAC1) activated kinase 2	XP_023479329	7.24	2.00	2.00	2.00	2.00	6.45	8.09	8.38	2.00	2.00	2.00	6.60	2.00	7.61	2.00	2.00
100053539	LAMB2	laminin subunit beta 2	3913	LAMB2	laminin subunit beta 2	XP_023476210	2.00	2.00	2.00	2.00	6.12	5.98	5.62	2.00	2.00	2.00	2.00	2.00	2.00	2.00	7.35	2.00
100069054	SEMA4B	semaphorin 4B	10509	SEMA4B	semaphorin 4B	XP_023505562	2.00	2.00	2.00	2.00	6.96	5.75	6.41	2.00	2.00	2.00	2.00	2.00	2.00	2.00	2.00	7.39
100630774	SPINK7	serine peptidase inhibitor Kazal type 7	84651	SPINK7	serine peptidase inhibitor, Kazal ty	XP_003362918	2.00	2.00	2.00	2.00	7.69	7.38	2.00	2.00	12.12	12.47	2.00	9.47	2.00	2.00	2.00	2.00
100054706	SEMA3C	semaphorin 3C	10512	SEMA3C	semaphorin 3C	XP_023494586	6.30	2.00	6.62	2.00	2.00	2.00	2.00	2.00	2.00	2.00	2.00	7.37	2.00	2.00	2.00	2.00
100066844	CDH1	cadherin 1	999	CDH1	cadherin 1	XP_023492960	2.00	2.00	2.00	2.00	2.00	7.47	6.73	2.00	2.00	2.00	2.00	2.00	2.00	2.00	2.00	2.00
100072339	ROCK2	Rho associated coiled-coil containing protein	9475	ROCK2	Rho associated coiled-coil contair	XP_023475297	4.90	2.00	2.00	2.00	4.86	2.00	4.81	2.00	2.00	2.00	2.00	2.00	2.00	2.00	2.00	2.00
100062401	SNX5	sorting nexin 5	27131	SNX5	sorting nexin 5	XP_023481842	2.00	2.00	2.00	2.00	6.19	6.30	2.00	2.00	2.00	2.00	2.00	2.00	2.00	2.00	2.00	5.49
111768608	KCTD12	potassium channel tetramerization domain cc	115207	KCTD12	potassium channel tetramerizatioi	XP_023477468	2.00	2.00	2.00	2.00	2.00	5.11	3.64	2.00	2.00	2.00	2.00	2.00	2.00	2.00	2.00	2.00
