# Supplementary figures and images for: Isolation and Characterization of Equine Uterine Extracellular Vesicles: A Comparative Methodological Study
Source: Int J Mol Sci. 2021 Jan 19;22(2):979. doi: 10.3390/ijms22020979 (PMC7835857; doi:10.3390/ijms22020979)

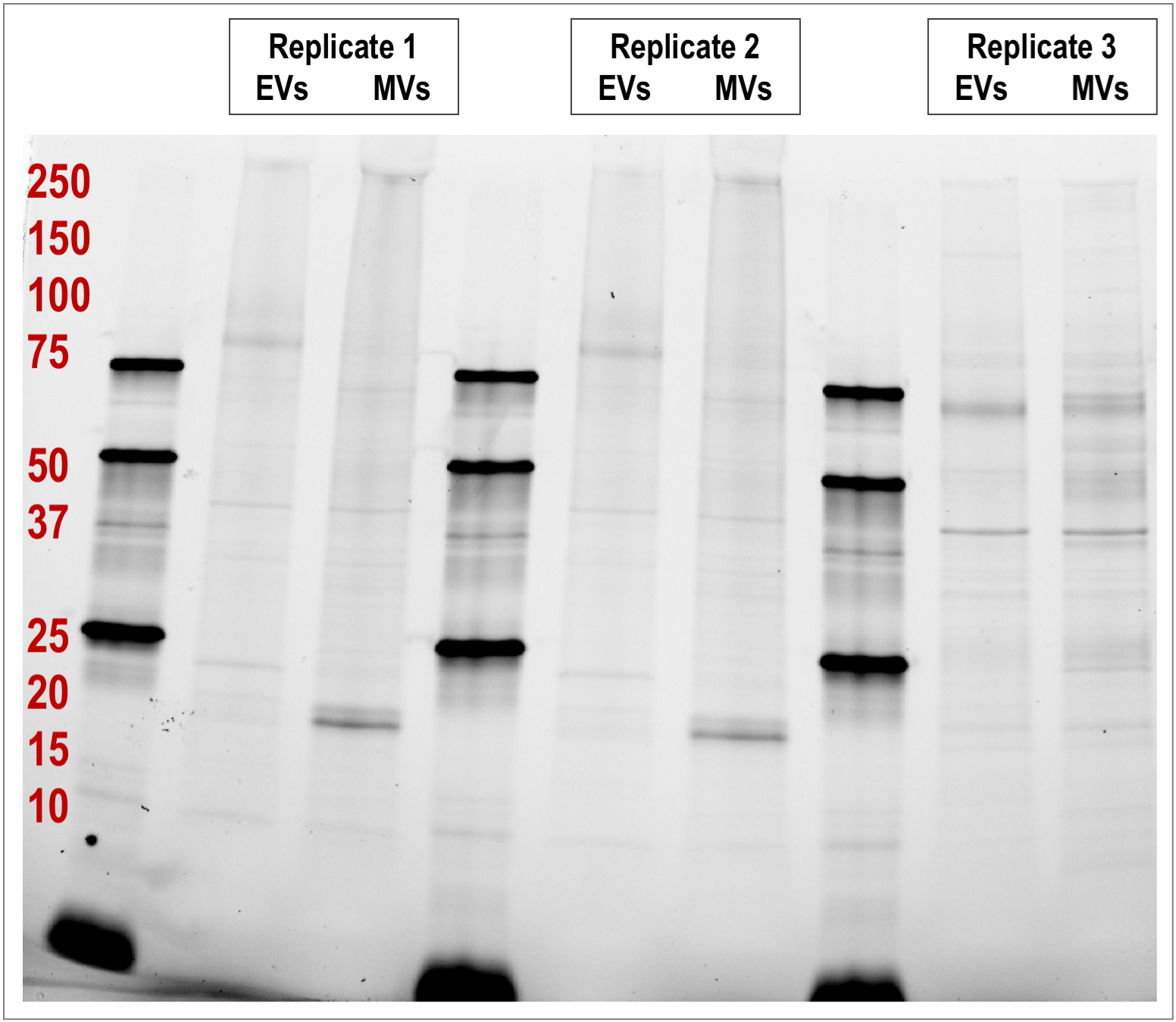

Supplement: Supplementary file 1 [file ijms-22-00979-s001.zip › Figure_S1.tif]

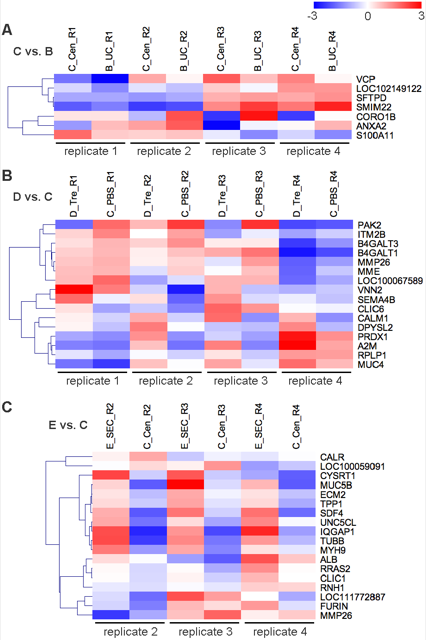

Supplement: Supplementary file 1 [file ijms-22-00979-s001.zip › Figure_S3.tif]

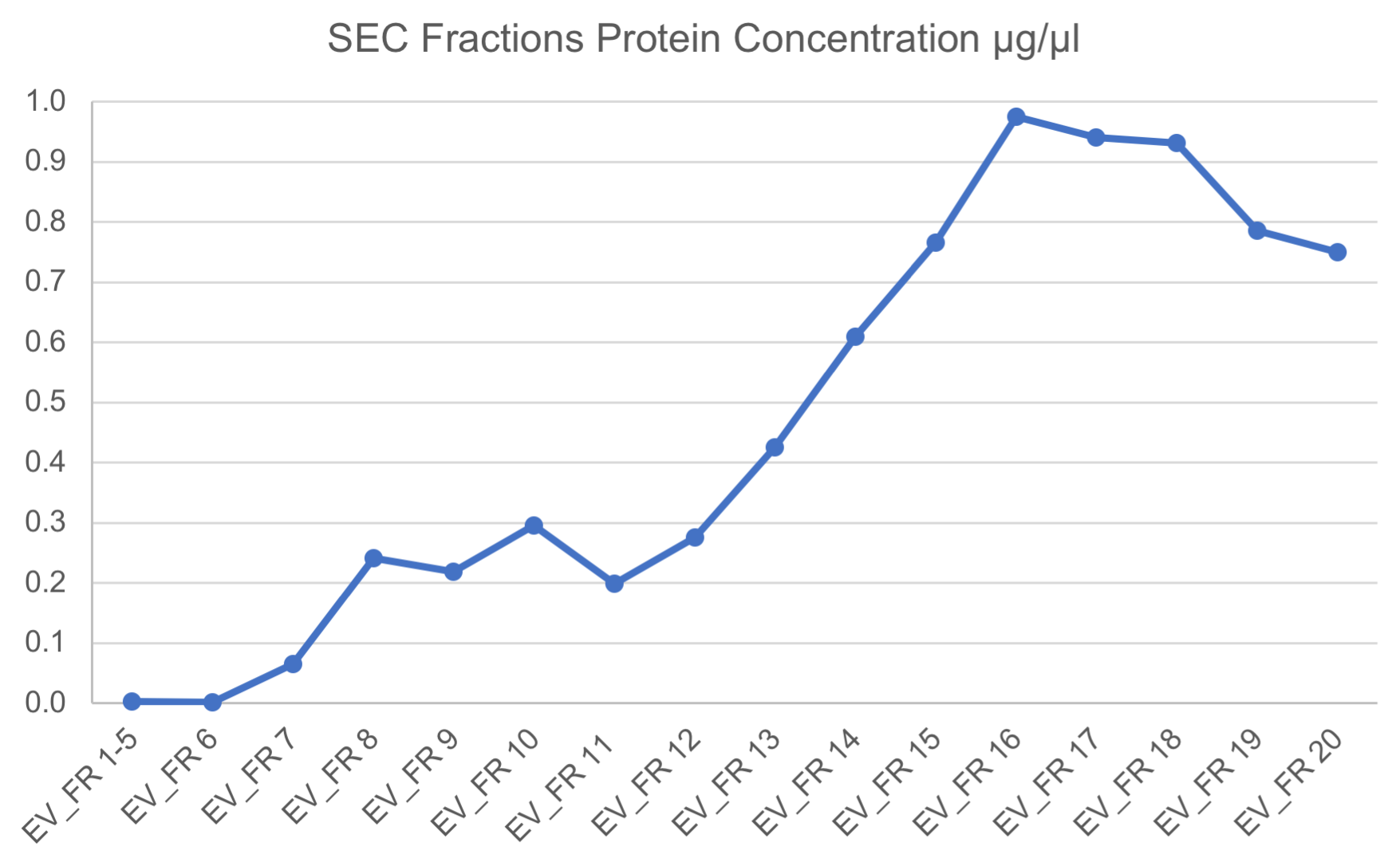

Supplement: Supplementary file 1 [file ijms-22-00979-s001.zip › Figure_S4.tif]
